# Adverse Outcomes in Obese Cardiac Surgery Patients Correlates With Altered Branched-Chain Amino Acid Catabolism in Adipose Tissue and Heart

**DOI:** 10.3389/fendo.2020.00534

**Published:** 2020-08-07

**Authors:** Dipsikha Biswas, Kathleen Tozer, Khoi T. Dao, Lester J. Perez, Angella Mercer, Amy Brown, Intekhab Hossain, Alexandra M. Yip, Christie Aguiar, Hany Motawea, Keith R. Brunt, Jennifer Shea, Jean F. Legare, Ansar Hassan, Petra C. Kienesberger, Thomas Pulinilkunnil

**Affiliations:** ^1^Department of Biochemistry and Molecular Biology, Dalhousie University, Dalhousie Medicine New Brunswick, Saint John, NB, Canada; ^2^IMPART Investigator Team Canada, Saint John, NB, Canada; ^3^New Brunswick Heart Centre, Saint John Regional Hospital, Saint John, NB, Canada; ^4^Department of Pharmacology, Dalhousie University, Dalhousie Medicine New Brunswick, Saint John, NB, Canada; ^5^Department of Pathology, Dalhousie University, Saint John, NB, Canada; ^6^Department of Laboratory Medicine, Saint John Regional Hospital, Saint John, NB, Canada

**Keywords:** BCAA, BCKDH, BCAA metabolism, obesity, subcutaneous adipose, atrial appendage, cardiac surgery

## Abstract

**Background:** Predicting relapses of post-operative complications in obese patients who undergo cardiac surgery is significantly complicated by persistent metabolic maladaptation associated with obesity. Despite studies supporting the linkages of increased systemic branched-chain amino acids (BCAAs) driving the pathogenesis of obesity, metabolome wide studies have either supported or challenged association of circulating BCAAs with cardiovascular diseases (CVDs).

**Objective:** We interrogated whether BCAA catabolic changes precipitated by obesity in the heart and adipose tissue can be reliable prognosticators of adverse outcomes following cardiac surgery. Our study specifically clarified the correlation between BCAA catabolizing enzymes, cellular BCAAs and branched-chain keto acids (BCKAs) with the severity of cardiometabolic outcomes in obese patients pre and post cardiac surgery.

**Methods:** Male and female patients of ages between 44 and 75 were stratified across different body mass index (BMI) (non-obese = 17, pre-obese = 19, obese class I = 14, class II = 17, class III = 12) and blood, atrial appendage (AA), and subcutaneous adipose tissue (SAT) collected during cardiac surgery. Plasma and intracellular BCAAs and BC ketoacids (BCKAs), tissue mRNA and protein expression and activity of BCAA catabolizing enzymes were assessed and correlated with clinical parameters.

**Results:** Intramyocellular, but not systemic, BCAAs increased with BMI in cardiac surgery patients. In SAT, from class III obese patients, mRNA and protein expression of BCAA catabolic enzymes and BCKA dehydrogenase (BCKDH) enzyme activity was decreased. Within AA, a concomitant increase in mRNA levels of BCAA metabolizing enzymes was observed, independent of changes in BCKDH protein expression or activity. BMI, indices of tissue dysfunction and duration of hospital stay following surgery correlated with BCAA metabolizing enzyme expression and metabolite levels in AA and SAT.

**Conclusion:** This study proposes that in a setting of obesity, dysregulated BCAA catabolism could be an effective surrogate to determine cardiac surgery outcomes and plausibly predict premature re-hospitalization.

## Introduction

Current estimates suggest that >35% of patients undergoing heart surgery are obese (body mass index (BMI) >30 kg/m^2^) (1). Obesity is causally linked to the development of IR and T2DM which increases the risk for cardio metabolic disorders (2–4) as well as susceptibility of re-hospitalization (5, 6). Predicting relapses of post-operative complications in obese patients who undergo cardiac surgery is significantly complicated by persistent metabolic maladaptation associated with obesity, such as increases in circulating lipids, glucose, adipocytokines (leptin, insulin, RBP4) and branch chain amino acids (BCAAs). Epidemiological studies have supported the association between circulating BCAAs (leucine, isoleucine and valine) and incidences of obesity (7, 8), insulin resistance (IR) (9, 10), type 2 diabetes mellitus (T2DM) (11–13). However, the relationship between systemic BCAAs and cardiovascular dysfunction (CVDs) are both supported and contradicted by numerous studies. For instance, circulating BCAAs were associated with greater prevalence of coronary disease (1, 14, 15), acute heart failure (HF) (16, 17) and cardiometabolic risk (18), specifically in the setting of T2DM and in most instances independent of BMI. In contrast, multiple metabolome wide studies of ischemic heart disease (19, 20) myocardial infarction (21) and incident coronary artery disease (22, 23) have failed to attribute elevated BCAAs for driving CVD. Besides, other groups have reported reduced circulating BCAA levels following myocardial infarction (24) and during stable coronary heart disease (25). Interestingly, in rodents (26, 27) and humans (28) exogenous BCAA supplementation attenuated heart failure complications, challenging the dogma that systemic BCAA elevation is causal for CVD. These inconsistencies in the association of circulating BCAA levels and CVDs in the absence of T2DM have necessitated studies focusing on BCAAs metabolic intermediates and tissue specific changes in BCAAs catabolizing enzymes in driving cardiac pathology.

Alterations in systemic BCAAs is an outcome of their dietary intake and effective intracellular catabolism (29, 30). BCAAs, unlike other amino acids, are primarily catabolized in extra hepatic tissues, such as cardiac muscle (31) and their effective utilization is critical to maintain normal cardiac function (32). Reversible transamination by branch chain aminotransferase (BCAT) initiates BCAA catabolism yielding branched-chain α-keto acids (BCKAs) followed by oxidative decarboxylation by branched-chain ketoacid dehydrogenase (BCKDH) (29). BCKDH is the rate limiting enzyme of the pathway, and its activity is sensitive to inhibitory phosphorylation by branched-chain keto acid dehydrogenase kinase (BCKDK) and is positively regulated by protein phosphatase 1K (PPM1K). Through a series of enzymatic reactions, BCKAs are converted into either succinyl CoA, acetyl CoA or propionyl CoA, which serve as TCA cycle intermediates vital for ATP production. Transcriptional programming of BCAA catabolism is regulated by Kruppel like factor 15 (KLF15) (33). Defective BCAA catabolism is reported in both human and rodent failing hearts (33–35) along with identification of KLF15 as the key regulator of cardiac BCAA metabolism (33). Recent studies have also demonstrated that independent of systemic changes of BCAA, cardiac tissues are highly susceptible to injury from dysregulated BCAA metabolism. Indeed, defective BCAA catabolism and accumulation of BCAAs renders the heart vulnerable to ischemic and hypertrophic injury (36, 37).

We questioned whether BCAA catabolic changes precipitated by obesity in the heart and adipose tissue, the two important tissue depots responsible for efficient utilization of BCAAs, can be reliable prognosticators of adverse outcomes following cardiac surgery. Our study aimed to clarify the correlation between cellular BCAAs and their catabolite levels along with the ability of individual tissues to metabolize BCAAs with the severity of cardiometabolic outcomes in obese patients pre and post cardiac surgery. This study also examined if metabolic status of tissue BCAAs is a robust and definitive predictor of morbidity, post-operative tissue dysfunction and length of stay in cardiac surgery patients with obesity.

## Results

### Patient Characteristics

Patient characteristics and pre-operative parameters have been listed in Table 1. Age at the time of surgery ranged from 44 to 75 years and was similar between BMI groups. Patients with a BMI of 18.5–24.9 kg/m^2^ were considered as the non-obese control group (*n* = 17), patients with a BMI of 25.0–29.9 kg/m^2^ were classified as pre-obese (*n* = 19), patients with a BMI of 30–34.9 kg/m^2^ were classified as class I obese (*n* = 14), patients with a BMI of 35.0–39.9 kg/m^2^ were classified as class II obese (*n* = 17) and patients with a BMI of ≥40 kg/m^2^ were classified as class III obese (*n* = 12). Concurrently, the mean waist circumference was 88.8, 102.19, 110.3, 124.3, and 138 cm for non-obese, pre-obese, and class I-III obese patients, respectively. Eighty-one percentage of our patient cohort were males. There was no significant difference between males and females between the non-obese and pre-obese (males = 30, females = 6) vs. obese (males = 34, females = 9) groups. Individuals with current or past smoking history comprised 69% of non-obese, 55% of pre-obese, 46% of class I obese, 56% of class II obese and 58% of class III obese patients undergoing cardiac surgery. Thirty percentage of our patient cohort were diabetic. In each BMI category, majority of patients were on beta-blockers, angiotensin-converting enzyme inhibitors and/or angiotensin receptor blockers for blood pressure management and taking lipid lowering statins. No significant differences were observed across the groups. Heart failure in NYHA Class 3 and 4 categories was evident preoperatively in 25% of non-obese, 35% of pre-obese, 31% of the class I obese, 44% of the class II obese and 75% of the class III obese patients. Due to unavoidable circumstances during patient recruitment, all the parameters listed in Table 1 could not be populated. All patients underwent elective heart surgery with 55.69% of cases being isolated CABG surgery, 20.25% isolated valve surgery, and 24.05% a combination of valve and CABG surgery. No significant differences were observed between different BMI groups as described in Table 1. Unadjusted Spearman correlations between post-operative clinical parameters with the mRNA and protein expression of BCAA catabolic enzymes have been summarized in Tables 2–5. Unadjusted Spearman correlations between pre- and post-operative clinical parameters with the BCKDH enzyme activity and intracellular BCKAs and BCAAs have been summarized in Table 6. Only those parameters that were significantly correlated with the BCAA enzyme expression and metabolite levels in SAT and AA have been included in the Tables 2–6. Spearman correlations for non-normally distributed variables between pre-operative parameters and with protein and mRNA levels of BCAA catabolic enzymes also revealed similar correlations (data not shown).

**Table 1:** Patient characteristics and metabolic parameters.

**Parameter**	**Non-obese**	**Pre-obese**	**Obese class I**	**Obese class II**	**Obese class III**	**Statistical analysis**
	***n* = 17**	***n* = 19**	***n* = 14**	***n* = 17**	***n* = 12**	
**BMI**	**18.5–24.9 kg/m**^**2**^	**25–29.9 kg/m**^**2**^	**30–34.9 kg/m**^**2**^	**35.0–39.9 kg/m**^**2**^	**≥40.0 kg/m**^**2**^	
**Demographic data**						
Male/Female	11/6	19/0	11/3	14/3	9/3	
Age (years)	64.25 (6.49)	62.6 (7.27)	65.23 (6.01)	64.75 (5.56)	62.0 (8.81)	ns across groups
Smoker (*n*)	11 (69)	11 (55)	6 (46)	9 (56)	7 (58)	
Medications						ns across all groups^∧^
Blood pressure-lowering (*n*)	8 (50)	13 (65)	8 (62)	12 (75)	10 (84)	
Beta blocker (*n*)	8 (50)	9 (45)	4 (31)	9 (45)	6 (50)	
ACE inhibitor (*n*)	12 (75)	9 (45)	8 (62)	10 (63)	7 (59)	
Angiotensin receptor blocker (*n*)	0 (0)	3 (15)	2 (16)	5 (32)	3 (25)	
Lipid lowering (*n*)	10 (63)	14 (70)	9 (69)	11 (69)	9 (75)	
**Parameters of obesity**						
BMI (kg/m^2^)	22.83 (1.24)	27.76 (1.48)	31.68 (1.12)	37.03 (1.61)	43.74 (3.73)	N vs. PO: ^****^
						N vs. OI: ^****^
						N vs. OII: ^****^
						N vs. OIII: ^****^
						PO vs. OI: ^****^
						PO vs. OII: ^****^
						PO vs. OIII: ^****^
						OI vs. OII: ^****^
						OI vs. OIII: ^****^
						OII vs. OIII: ^****^
Waist circumference (cm)	88.80 (9.17)	102.19 (7.84)	110.3 (6.29)	124.3 (8.83)	138.0 (12.76)	N vs. PO: ^***^
						N vs. OI: ^****^
						N vs. OII: ^****^
						N vs. OIII: ^****^
						PO vs. OI: ns
						PO vs. OII: ^****^
						PO vs. OIII: ^****^
						OI vs. OII: ^**^
						OI vs. OIII: ^****^
						OII vs. OIII: ^**^
Hip circumference (cm)	97.25 (4.05)	102.03 (3.83)	108.4 (5.96)	118.3 (10.03)	132.0 (10.54)	N vs. PO: ns
						N vs. OI: ^**^
						N vs. OII: ^****^
						N vs. OIII: ^****^
						PO vs. OI: ns
						PO vs. OII: ^****^
						PO vs. OIII: ^****^
						OI vs. OII: ^**^
						OI vs. OIII: ^****^
						OII vs. OIII: ^****^
**Metabolic parameters**						
Hypertension (*n*)	8 (50)	12 (60)	7 (53.8)	11 (68.75)	10 (83.33)	
Triglycerides fasting (mmol/L)	1.28 (0.43)	1.45 (1.40)	1.55 (0.41)	2.09 (0.88)	1.63 (0.57)	ns across groups
HDL cholesterol fasting (mmol/L)	1.40 (0.30)	1.23 (0.38)	1.18 (0.28)	1.09 (0.4)	1.16 (0.44)	ns across groups
Cholesterol fasting (mmol/L)	4.33 (0.83)	4.18 (1.49)	3.66 (0.58)	4.03 (0.9)	3.88 (1.08)	ns across groups
LDL cholesterol fasting (mmol/L)	2.36 (0.82)	2.196 (1.06)	1.77 (0.56)	2.08 (0.73)	1.86 (0.78)	ns across groups
**NYHA Classification**						
1 (*n*)	2 (12.5)	6 (30)	2 (15.4)	1 (6.3)	0 (0)	
2 (*n*)	7 (43.8)	5 (25)	4 (31)	5 (31.2)	1 (8.3)	
3 (*n*)	3 (18.8)	4 (20)	3 (23)	7 (43.7)	6 (50)	
4 (*n*)	1 (6.3)	3 (15)	1 (7.7)	2 (12.5)	3 (25)	
Diabetes mellitus type 2 (*n*)	2 (12.5)	8 (40)	4 (30.77)	4 (25)	6 (50)	
Glucose random blood (mmol/L)	6.07 (3.43)	7.85 (3.63)	7.77 (3.38)	7.49 (2.62)	8.85 (2.98)	ns across groups
Procedure						ns across all groups^#^
Isolated CABG	9 (52.94)	14 (73.68)	8 (57.14)	9 (52.94)	4 (33.33)	
Isolated valve	2 (11.7)	3 (15.78)	3 (21.42)	3 (17.64)	5 (41.66)	
Combined	6 (35.29)	2 (10.52)	3 (21.42)	5 (29.41)	3 (25)	
Other	4 (23.53)	0 (0)	1 (7.14)	1 (5.88)	2 (16.78)	

Data are expressed as mean (standard deviation) for continuous variables and number (percent of population) for categorical values, as described previously [70]. One-way ANOVA followed by a Tukey post hoc test was performed for the statistical analysis. ^∧^Chi square goodness of fit test was performed for statistical analysis of the medications for our patient cohort (p = 0.965, Chi square = 7.362);

# Chi square goodness of fit test was performed for statistical analysis of the surgery procedures (p = 0.395, Chi square = 8.394). N, non-obese; PO, pre-obese; OI, obese class I; OII, obese class II; OIII, obese class III; ns, non-significant;

** P < 0.01,

*** P < 0.001,

**** *P < 0.0001*.

**Table 2:** Unadjusted Spearman's correlations for protein expression data of BCAA pathway enzyme in SAT from cardiac surgery patients.

**Variable**	**BCKDH**	**pBCKDE1α**	**BCKDK**	**KLF15**	**SOAT1**	**DLD**	**ERAB**
**Parameters of obesity**							
Waist circumference	−0.29786	**0.49228**	−0.03234	−0.23354	0.35835	0.03727	−0.12754
	*0.2021*	***0.0275***	*0.8923*	0.2509	0.0722	0.8566	*0.5347*
Hip circumference	−0.41444	0.38728	−0.15495	−0.20212	**0.57250**	0.01710	−0.23358
	*0.0692*	*0.0916*	*0.5142*	*0.3221*	***0.0022***	*0.9339*	*0.2508*
**Metabolic parameters**							
Fasting Insulin	−0.42857	**0.62198**	−0.23956	−0.12331	0.21805	0.05414	−0.31128
	*0.1263*	***0.0176***	*0.4094*	*0.6045*	*0.3557*	*0.8207*	*0.1816*
**Blood chemistry**							
Hemoglobin	0.01627	−0.19779	−0.26823	0.17462	–**0.49633**	−0.14312	−0.01437
	*0.9442*	*0.3901*	*0.2397*	*0.3837*	***0.0085***	*0.4764*	*0.9433*
Leukocytes	0.09165	0.28195	0.04420	–**0.38113**	0.09253	−0.06993	0.17132
	*0.6928*	*0.2156*	*0.8491*	***0.0498***	*0.6462*	*0.7289*	*0.3929*
Erythrocytes	0.03774	−0.19206	–**0.56344**	−0.14240	−0.28694	−0.28938	−0.11856
	*0.8710*	*0.4043*	***0.0078***	*0.4786*	*0.1467*	*0.1432*	*0.5559*
Leucine	−0.29772	−0.42732	−0.07706	0.35744	0.02686	–**0.54959**	0.25310
	*0.3473*	*0.1659*	*0.8119*	*0.1453*	*0.9157*	***0.0181***	*0.3109*
**Echocardiography/angiography**							
Left ventricular end diastolic pressure	−0.15339	0.35666	0.25031	−0.09719	**0.45599**	0.28779	−0.18129
	*0.5185*	*0.1227*	*0.2871*	*0.6367*	***0.0192***	*0.1540*	*0.3754*

*Data for each correlation are expressed as Spearman's r (top) followed by P-value (bottom, italic), denoted in bold when P < 0.05*.

**Table 3:** Unadjusted Spearman's correlations for mRNA expression data of BCAA pathway enzyme in SAT from cardiac surgery patients.

**Variable**	**BCKDHA**	**BCKDHB**	**BCKDK**	**PPM1K**	**KLF15**	**ACADSB**	**HADHA**
**Parameters of obesity**							
Waist circumference	−0.12793	−0.05028	0.15152	–**0.52108**	−0.20358	−0.33819	−0.19137
	*0.5005*	*0.7919*	*0.4241*	***0.0032***	*0.2806*	*0.0676*	*0.3111*
Hip circumference	−0.30234	−0.09455	−0.01758	–**0.63693**	−0.21468	–**0.52436**	–**0.45478**
	*0.1044*	*0.6192*	*0.9266*	***0.0002***	*0.2546*	***0.0029***	***0.0116***
**Metabolic parameters**							
Triglycerides fasting	0.00556	0.32102	0.19422	–**0.40957**	−0.16174	−0.03270	0.10235
	*0.9767*	*0.0837*	*0.3038*	***0.0246***	*0.3932*	*0.8638*	*0.5905*
Cholesterol fasting	0.07142	**0.44899**	0.15975	0.12237	0.16242	−0.01268	0.11927
	*0.7076*	***0.0128***	*0.3991*	*0.5194*	*0.3912*	*0.9470*	*0.5302*
LDL cholesterol fasting	0.14597	**0.37784**	0.14709	0.29684	0.22675	0.02715	0.19539
	*0.4415*	***0.0395***	*0.4380*	*0.1112*	*0.2282*	*0.8868*	*0.3008*
**Blood chemistry**							
Blood urea	−0.06442	–**0.44494**	−0.20508	−0.02452	−0.11458	0.08805	0.07056
	*0.7352*	***0.0138***	*0.2770*	*0.8977*	*0.5466*	*0.6436*	*0.7110*
Leucine	−0.17370	0.13233	−0.08959	–**0.42845**	−0.38331	−0.13883	0.09267
	*0.3961*	*0.5193*	*0.6634*	***0.0290***	*0.0532*	*0.4988*	*0.6525*

*Data for each correlation are expressed as Spearman's r (top) followed by P-value (bottom, italic), denoted in bold when P < 0.05*.

**Table 4:** Unadjusted Spearman's correlations for protein expression data of BCAA pathway enzyme in AA from cardiac surgery patients.

**Variable**	**BCKDH**	**pBCKDE1α**	**BCKDK**	**BCAT2**	**KLF15**	**PPM1K**	**DLD**	**HIBCH**	**SOAT1**	**ERAB**
**Metabolic parameters**										
Triglycerides fasting	−0.12310	**0.51291**	−0.25201	−0.12151	−0.29003	0.11418	–**0.41398**	−0.28352	−0.28352	0.04824
	*0.5491*	***0.0074***	*0.2142*	*0.5460*	*0.1422*	*0.5707*	***0.0318***	*0.3260*	*0.3260*	*0.8112*
hsCRP	0.20000	0.53636	−0.02727	−0.22727	–**0.70000**	0.13636	0.00909	−0.67857	−0.53571	0.51818
	*0.5554*	*0.0890*	*0.9366*	*0.5015*	***0.0165***	*0.6893*	*0.9788*	*0.0938*	*0.2152*	*0.1025*
**Blood chemistry**										
Blood urea	**0.41684**	0.10815	0.25394	**0.43324**	−0.29881	**0.41094**	0.21632	0.22442	0.42244	−0.04675
	***0.0341***	*0.5990*	*0.2106*	***0.0240***	*0.1300*	***0.0332***	*0.2785*	*0.4405*	*0.1324*	*0.8169*
Hemoglobin	**0.39904**	0.11886	0.10413	−0.02141	−0.11835	−0.07615	−0.11835	0.37749	0.36424	−0.24434
	***0.0434***	*0.5631*	*0.6127*	*0.9156*	*0.5566*	*0.7058*	*0.5566*	*0.1833*	*0.2004*	*0.2193*
Leukocytes	−0.18745	**0.45220**	–**0.45322**	−0.26599	−0.10017	–**0.45198**	–**0.53565**	–**0.56044**	–**0.56923**	−0.08307
	*0.3592*	***0.0204***	***0.0201***	*0.1799*	*0.6191*	***0.0179***	***0.0040***	***0.0371***	***0.0336***	*0.6804*
Erythrocytes	0.20058	0.13247	−0.22557	−0.14790	−0.17448	–**0.42445**	−0.17448	0.17181	0.15639	−0.16226
	*0.3259*	*0.5189*	*0.2679*	*0.4616*	*0.3841*	***0.0273***	*0.3841*	*0.5570*	*0.5934*	*0.4187*
Neutrophils	−0.14660	0.35314	–**0.39801**	−0.15777	−0.14035	–**0.42624**	–**0.46476**	−0.34476	−0.31382	−0.08714
	*0.4748*	*0.0768*	***0.0440***	*0.4319*	*0.4850*	**0.0266**	***0.0146***	*0.2274*	*0.2746*	*0.6656*
Leucine	0.15828	0.11902	0.36074	0.32541	−0.31095	**0.48967**	0.29442	0.17857	0.03571	0.06612
	*0.5440*	*0.6491*	*0.1549*	*0.1876*	*0.2091*	***0.0391***	*0.2356*	*0.7017*	*0.9394*	*0.7944*
Alanine	–**0.50399**	0.05150	−0.40343	−0.28306	0.15289	−0.25310	−0.40186	−0.46429	−0.64286	−0.26136
	***0.0391***	*0.8444*	*0.1083*	*0.2550*	*0.5447*	*0.3109*	*0.0983*	*0.2939*	*0.1194*	*0.2948*
Glycine	−0.26274	0.06753	−0.33272	−0.22532	0.14677	−0.10129	−0.08992	–**0.89286**	–**0.92857**	**0.49302**
	*0.3083*	*0.7968*	*0.1919*	*0.3687*	*0.5611*	*0.6892*	*0.7227*	***0.0068***	***0.0025***	***0.0376***
Aspartic acid	−0.00736	0.10429	−0.12025	−0.37810	−0.02376	−0.00930	−0.21694	–**0.82143**	−0.75000	−0.06508
	*0.9776*	*0.6904*	*0.6457*	*0.1218*	*0.9254*	*0.9708*	*0.3872*	***0.0234***	*0.0522*	*0.7975*
**Echocardiography/angiography**										
Left ventricular end diastolic pressure	−0.31188	−0.05780	−0.23818	−0.19370	0.24368	−0.22472	−0.15717	–**0.58639**	−0.27215	0.23609
	*0.1291*	*0.7838*	*0.2516*	*0.3431*	*0.2303*	*0.2697*	*0.4432*	***0.0352***	*0.3684*	*0.2456*

*Data for each correlation are expressed as Spearman's r (top) followed by P-value (bottom, italic), denoted in bold when P < 0.05*.

**Table 5:** Unadjusted Spearman's correlations for mRNA expression data of BCAA pathway enzyme in AA from cardiac surgery patients.

**Variable**	**BCKDHA**	**BCKDHB**	**BCKDK**	**BCAT2**	**PPM1K**	**KLF15**	**ACADSB**	**HADHA**
**Parameters of obesity**								
Waist circumference	**0.48526**	0.16822	0.26366	0.30504	0.20736	0.08522	**0.41851**	**0.55045**
	***0.0066***	*0.3742*	*0.1592*	*0.1012*	*0.2715*	*0.6544*	***0.0214***	***0.0016***
Hip circumference	**0.44783**	0.23117	0.33437	0.17108	0.35039	0.14349	**0.43137**	**0.50167**
	***0.0131***	*0.2190*	*0.0709*	*0.3660*	*0.0577*	*0.4494*	***0.0173***	***0.0047***
**Metabolic parameters**								
hsCRP	**0.41769**	**0.50048**	**0.56385**	0.18615	0.20923	−0.14846	0.22308	0.17692
	***0.0377***	***0.0108***	***0.0033***	*0.3730*	*0.3155*	*0.4788*	*0.2838*	*0.3975*
**Blood chemistry**								
Blood urea	**0.38698**	**0.41601**	**0.41685**	0.03165	0.16808	−0.24877	0.12216	0.02430
	***0.0346***	***0.0222***	***0.0219***	*0.8681*	*0.3746*	*0.1850*	*0.5202*	*0.8986*
**Echocardiography/angiography**								
Left ventricular end diastolic pressure	−0.16139	−0.31241	−0.11453	−0.01531	–**0.43118**	−0.21834	−0.18098	−0.16598
	*0.4213*	*0.1126*	*0.5695*	*0.9396*	***0.0247***	*0.2739*	*0.3663*	*0.4080*

*Data for each correlation are expressed as Spearman's r (top) followed by P-value (bottom, italic), denoted in bold when P < 0.05*.

**Table 6:** Unadjusted Spearman's correlations (*p* < 0.05) for intracellular BCAA and BCKAs content and BCKDH enzyme activity in AA and SAT from cardiac surgery patients.

**Variables (AA)**	**KIV/Valine**	**KIC/Leu**	**KMV/Ile**	**BCKA/BCAA**	**KMV**	**KIV**	**Actual activity**
BMI	−0.4486	−0.4841	−0.6037	−0.5256	−0.4052	–	–
	*0.032*	*0.019*	*0.002*	*0.009*	*0.049*		
Waist Circumference	−0.3687	−0.4142	−0.4384	−0.4523	−0.3927	–	−0.4109
	*0.083*	*0.049*	*0.036*	*0.030*	*0.058*		*0.022*
Hip Circumference	–	−0.5114	−0.5258	−0.4892	−0.4648	–	−0.3616
		*0.013*	*0.009*	*0.002*	*0.022*		*0.046*
Length of Stay	–	–**0.4043**	−0.4757	–**0.3508**	−0.4106	–	–
		***0.055***	*0.021*	***0.08***	*0.046*		
NYHA	−0.4877	–	–	–	–	−0.4555	–
	*0.040*					*0.049*	
	**Valine**	**Isoleucine**	**Leucine**	**BCAA**	**Actual activity**		
HDL	−0.4320	−0.4511	−0.4405	−0.4137	–		
	*0.039*	*0.031*	*0.035*	*0.049*			
Random Blood Glucose	**0.3507**	**0.3474**	**0.3575**	**0.3405**	−0.4261		
	***0.082***	***0.075***	***0.093***	***0.070***	*0.015*		
**Variables (SAT)**	**Actual activity**	**Total activity**	**KIV**	**KIC**	**KMV**	**BCKAs**	
Random Blood Glucose	−0.3330	–**0.2922**	–	–	–	–	
	*0.044*	***0.079***					
Length of Stay	−0.3775	−0.4892	–	–	–	–	
	*0.021*	*0.002*					
Creatinine	–	–	–	−0.4226	−0.4638	–**0.3955**	
				*0.039*	*0.022*	***0.056***	
P-T BCKDH	−0.4585	−0.4179	–	–	–	–	
	*0.028*	*0.047*					
BCKDH	–	–	–**0.4506**	–	–	–	
			***0.079***				
DLD	**0.3814**	–	−0.5081	–	–	–	
	***0.072***		*0.044*				

*Data for each correlation are expressed as Spearman's r (top) followed by P-value (bottom, italic), denoted in bold when P is approaching significance*.

### Enzymes Catabolizing BCAAs Are Downregulated in SAT and Are Associated With BMI and Insulin Resistance in Obese Patients Undergoing Cardiac Surgery

**Figure 1:**
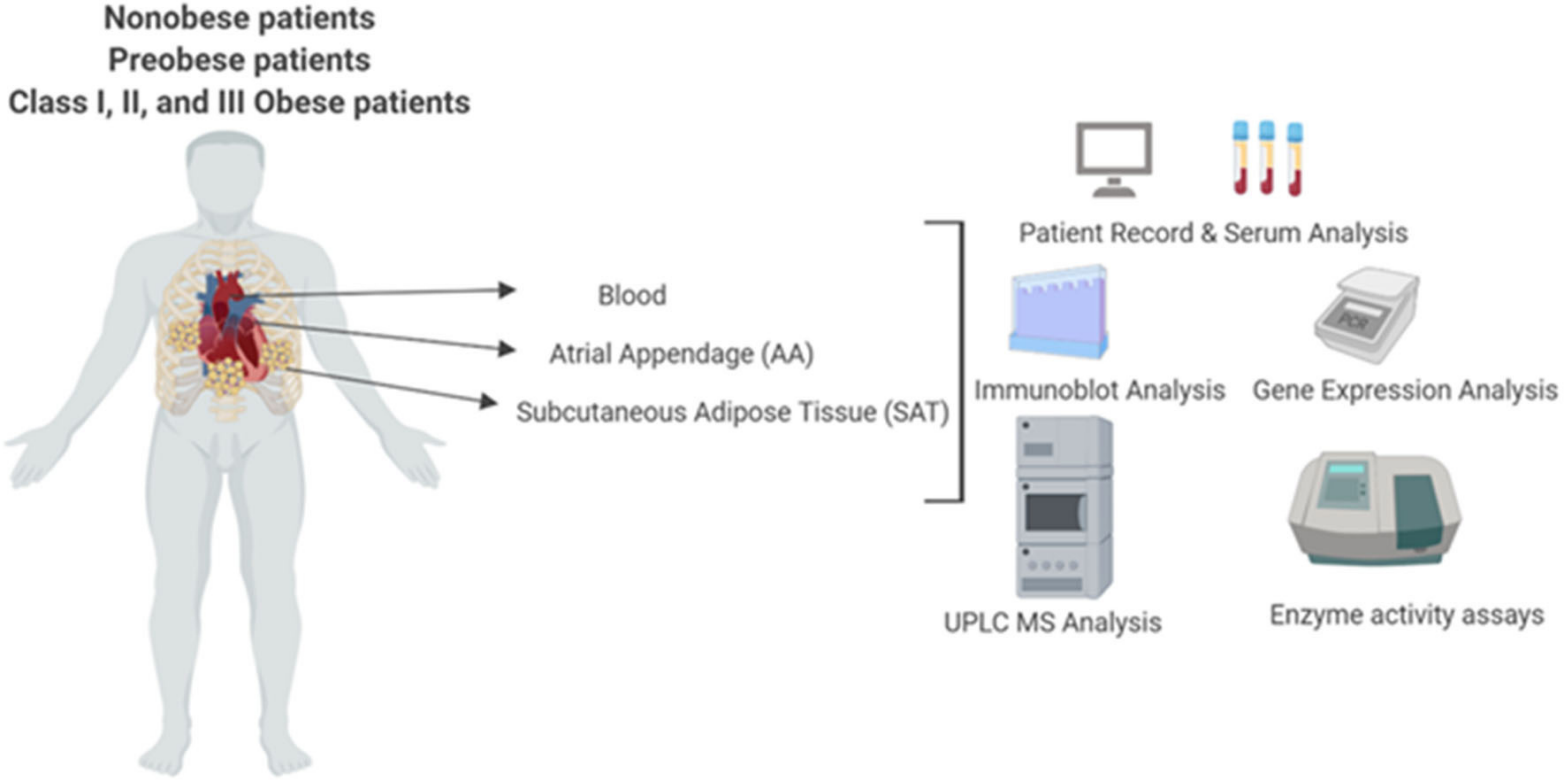
Experimental layout of UPLC-MS analysis of systemic and intracellular BCAA and BCKA measurements in the plasma, atrial appendage and subcutaneous adipose, tissue, BCAA catabolic enzyme mRNA and protein quantification and BCKDH enzyme activity measurements in heart and subcutaneous adipose tissue from non-obese, pre-obese, and obese patients undergoing cardiac surgery.

**Figure 2:**
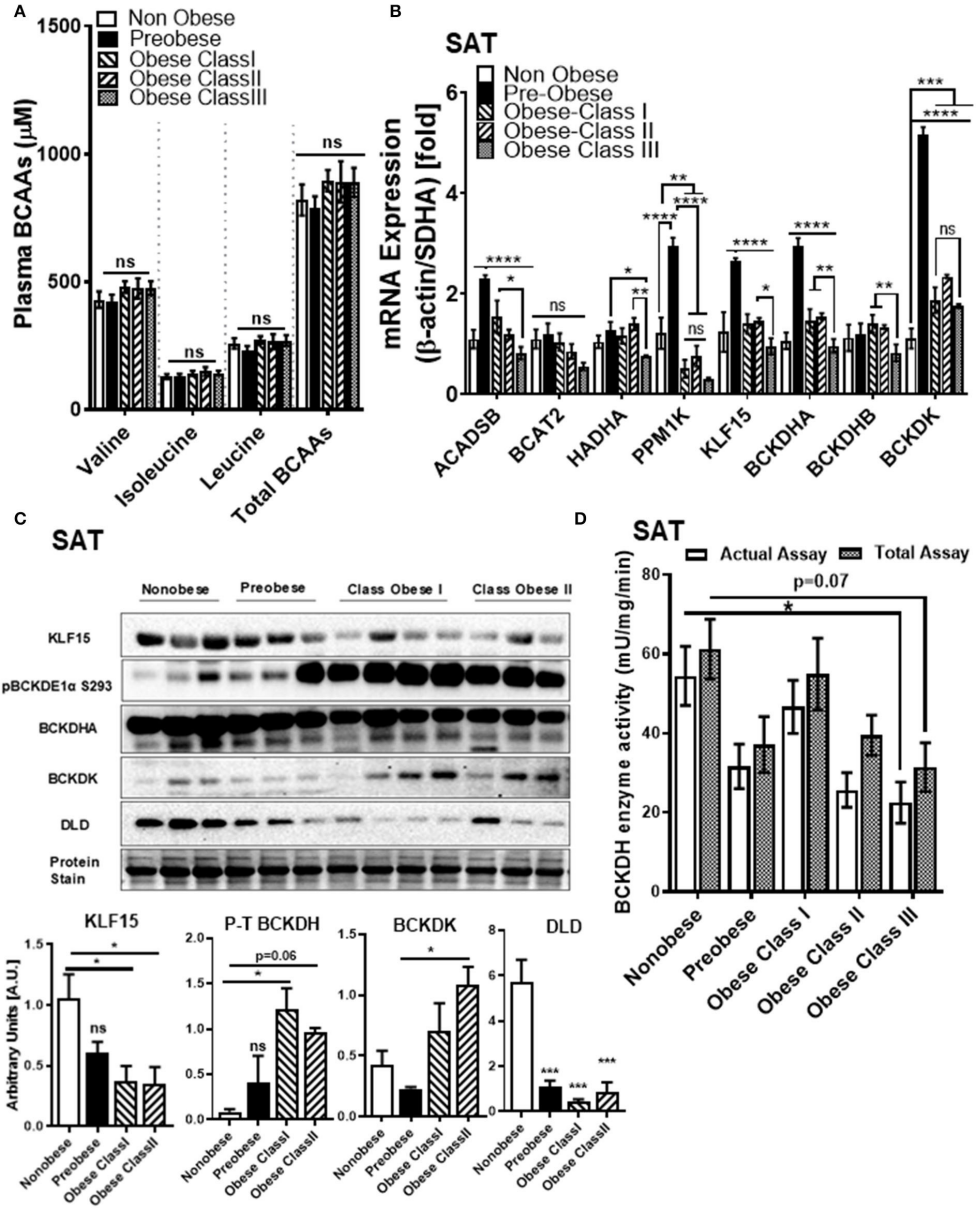
BCAA catabolic enzyme expression and BCKDH enzyme activity is decreased in the SAT of cardiac surgery patients with severe obesity. **(A)** Free plasma BCAA levels measured by UPLC MSMS in the non-obese (*n* = 8), pre-obese (*n* = 6) and class I obese (*n* = 7), class II obese (*n* = 6) and class III obese (*n* = 6) cardiac surgery patients. **(B)** Quantification of *ACADSB, BCAT2,HADHA, PPM1K, KLF15, BCKDHA, BCKDHB*, and *BCKDK* mRNA expression corrected to β*-actin* and *SDHA* (39) and expressed as fold change in the SAT of non-obese (*n* = 6), pre-obese (*n* = 6) and obese class I (*n* = 6), class II (*n* = 6) and class III (*n* = 6) cardiac surgery patients. **(C)** Representative immunoblot and densitometric analysis of KLF15, pBCKDE1a, BCKDH, BCKDK, and DLD protein levels in the SAT of non-obese (*n* = 3), pre-obese (*n* = 3), class I obese (*n* = 4) and class II obese (*n* = 3) cardiac surgery patients. **(D)** Total and actual BCKDH enzyme activity corrected to protein levels, measured at Vmax (*t* = 15 min) in the SAT of non-obese (*n* = 7), pre-obese (*n* = 9), class I obese (*n* = 9), class II obese (*n* = 9) and class III obese (*n* = 8). Statistical analysis was performed using a two-way ANOVA followed by a Tukey's multiple comparison test; *p < 0.05, **p < 0.01, ***p < 0.001, ****p < 0.0001 as indicated (40).

Despite strong linkages between BCAA and T2DM, the relationship between systemic BCAA levels and their ability to precipitate CVD in obese and diabetic patients is inconsistent (38). To examine the status of systemic BCAAs and its metabolic intermediates in our patient cohort, we measured circulating BCAAs and BCKAs in the plasma of cardiac surgery patients with different classes of obesity (Figure 1). Our study did not reveal significant differences in plasma BCAA levels across the different BMI groups in the cardiac surgery patients (Figure 2A). To investigate whether tissue specific alterations in the BCAA catabolic enzymes could be a determining factor specifically during the progression of obesity in patients undergoing cardiac surgery, we determined the mRNA and protein levels of key BCAA catabolic enzymes (Supplementary Figure 1, highlighted by ^*^) in SAT, an important tissue impacting BCAA catabolism (41). mRNA expression of BCAA catabolic enzymes in SAT were progressively reduced with increasing severity of obesity (Figure 2B). Along with the key BCAA catabolic enzymes, namely, BCAT2 (branched-chain amino transferase 2), *ACADSB* (short/branched-chain acyl CoA dehydrogenase), *HADHA* (hydroxyacyl CoA dehydrogenase, alpha subunit), *BCKDH A* and *B* subunits, the transcriptional regulator *KLF15* was also downregulated in class III obese patients (Figure 2B). Concomitantly, the inhibitory kinase, *BCKDK*, was augmented in the obese patients (compared to non-obese) whereas, activating phosphatase *PPM1K* (protein phosphatase Mg^2+^/Mn^2+^ phosphatase) levels were reduced in morbid obesity (compared to both non-obese and pre-obese patients) (Figure 2B). Our data indicate that in the SAT of class III obese cardiac surgery patients, BCAA catabolizing enzymes are downregulated. Alterations in mRNA expression of enzymes involved in BCAA metabolism were particularly pronounced in the obese group when compared to the pre-obese group of cardiac surgery patients. Protein expression of KLF15 was reduced in the SAT of obese class I and class II patients (Figure 2C). Expression of dihydrolipoamide dehydrogenase (DLD), the E3 regulating enzyme of the BCKDH complex, was decreased in the pre-obese, class I and class II obese patients. Furthermore, a decrease in the expression of BCKDH subunit A was observed in class II obese patients undergoing cardiac surgery (Figure 2C). A marked increase in BCKDK protein expression with a corresponding increase in the inhibitory phosphorylated BCKDH E1 (pBCKDE1) was observed in the SAT of class I and class II obese patients (Figure 2C). Both the actual and total BCKDH activity in the SAT depicted a decreasing trend with increasing obesity, with this reduction reaching statistical significance in class III obese cardiac surgery patients (Figure 2D, Supplementary Figure 2A). These findings highlight the downregulation of mRNA, protein as well as activity of BCAA catabolic enzymes in the SAT in close proximity to the heart, of cardiac surgery patients with obesity.

**Figure 3:**
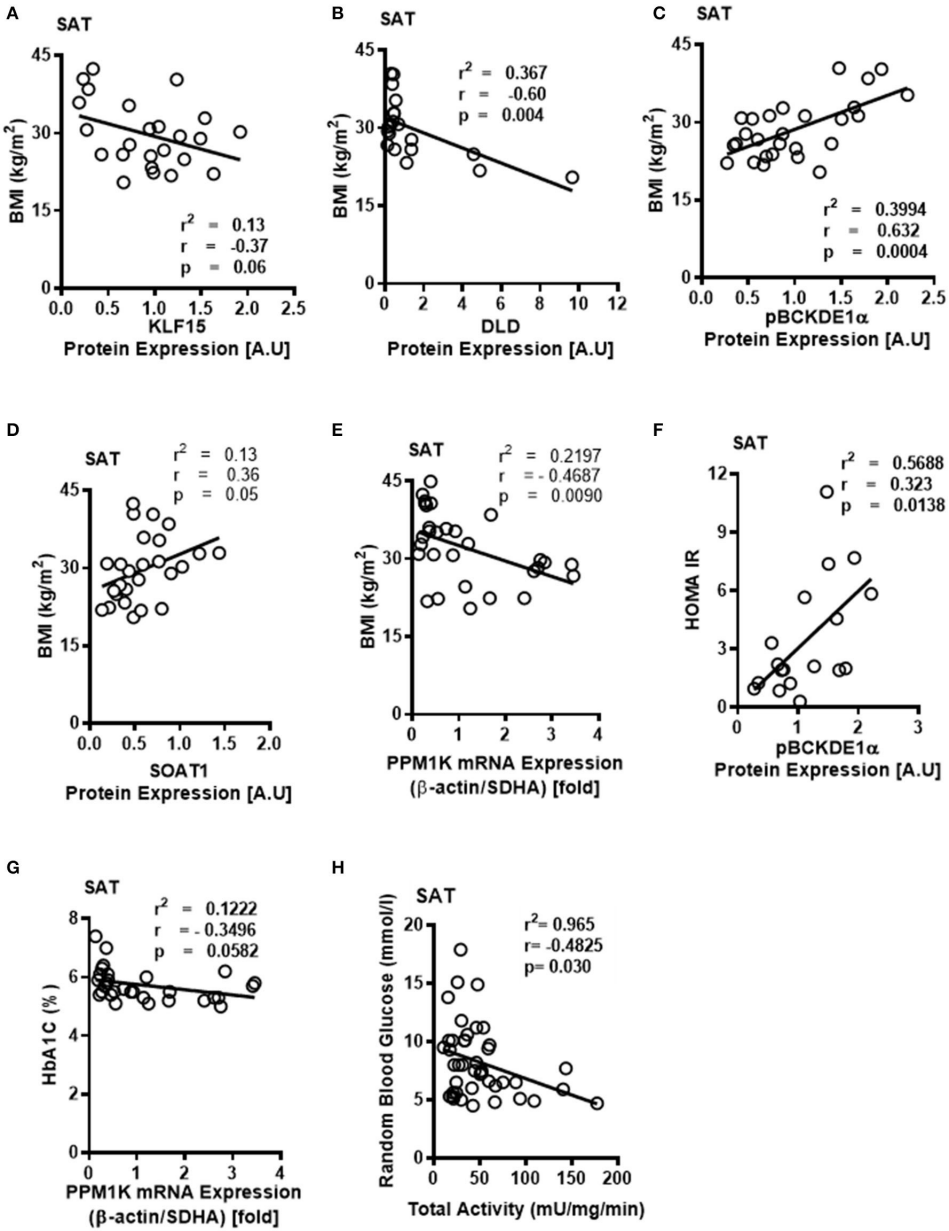
Downregulated BCKDH enzyme activity and expression of BCAA catabolic enzymes in the SAT of obese patients are associated with BMI and markers of insulin resistance. Linear regression of **(A)** KLF15, **(B)** DLD, **(C)** phosphorylated BCKDE1a, **(D)** SOAT1 protein expression in the SAT of non-obese (*n* = 7), pre-obese (*n* = 7), class I obese (*n* = 8) and class II obese (*n* = 6) cardiac surgery patients and **(E)** *PP1MK* mRNA expression in the SAT of non-obese (*n* = 6), pre-obese (*n* = 6), class I obese (*n* = 6), class II obese (*n* = 6) and class III obese (*n* = 6) cardiac surgery patients correlated with BMI. Linear regression of **(F)** pBCKDE1α protein levels in the SAT of non-obese (*n* = 7), pre-obese (*n* = 7), class I obese (*n* = 8) and class II obese (*n* = 6), **(G)** *PP1MK mRNA* levels in the SAT of non-obese (*n* = 6), pre-obese (*n* = 6), class I obese (*n* = 6), class II obese (*n* = 6) and class III obese (*n* = 6) and **(H)** Total BCKDH enzyme activity in the SAT of non-obese (*n* = 7), pre-obese (*n* = 9), class I obese (*n* = 9), class II obese (*n* = 9) and class III obese (*n* = 8) correlated with HOMA-IR,HbA1c% and random blood glucose levels, respectively, in the SAT of cardiac surgery patients. Statistical analysis was performed using one-way ANOVA; followed by a Tukey's multiple comparison test; *p* < 0.05 was considered significant.

Notably, downregulation of SAT KLF15 (Figure 3A) and DLD (Figure 3B) and upregulation of SAT pBCKDE1 levels (Figure 3C) were correlated with BMI; with pBCKDE1 levels also being positively correlated with hip circumference (Table 2). However, protein levels of SOAT1/ACAT1 (acetyl CoA acetyl transferase 1), an important enzyme of the isoleucine degradation pathway, was found to be positively correlated with BMI (Figure 3D). Furthermore, mRNA levels of *PPM1K* was negatively correlated with BMI (Figure 3E) as well as with parameters of obesity, like hip and waist circumference (Table 3). Similarly, reduced *ACADSB* and *HADH* mRNA levels were also correlated with increased hip circumference (Table 3), suggesting that in the SAT, BCAA catabolic enzymes are downregulated in the setting of obesity.

Obesity induces IR (42) and intraoperative IR and could influence cardiac surgery outcomes either due to increased risk of T2DM or independent of the patient's diabetic state (43). We examined whether the BCAA catabolic enzyme defects are associated with fasting blood glucose and fasting insulin levels as well as HbA1c and HOMA-IR, markers of IR, in obese patients. Our findings demonstrated a correlation between HOMA-IR (Figure 3F), fasting insulin (Table 2) and increased inhibitory phosphorylation of BCKDH E1 subunit (pBCKDE1) in SAT of obese patients. Alternatively, *PPM1K* mRNA levels tended to negatively correlate with HbA1C% in the SAT (Figure 3G); while both the actual and total BCKDH activity tended to be negatively correlated with the random blood glucose levels (Figure 3H).

### Correlation of BMI With Increased mRNA Expression of BCAA Catabolizing Enzymes as Well as Elevated Intramyocellular BCAAs in AA of Patients With Obesity Undergoing Cardiac Surgery

**Figure 4:**
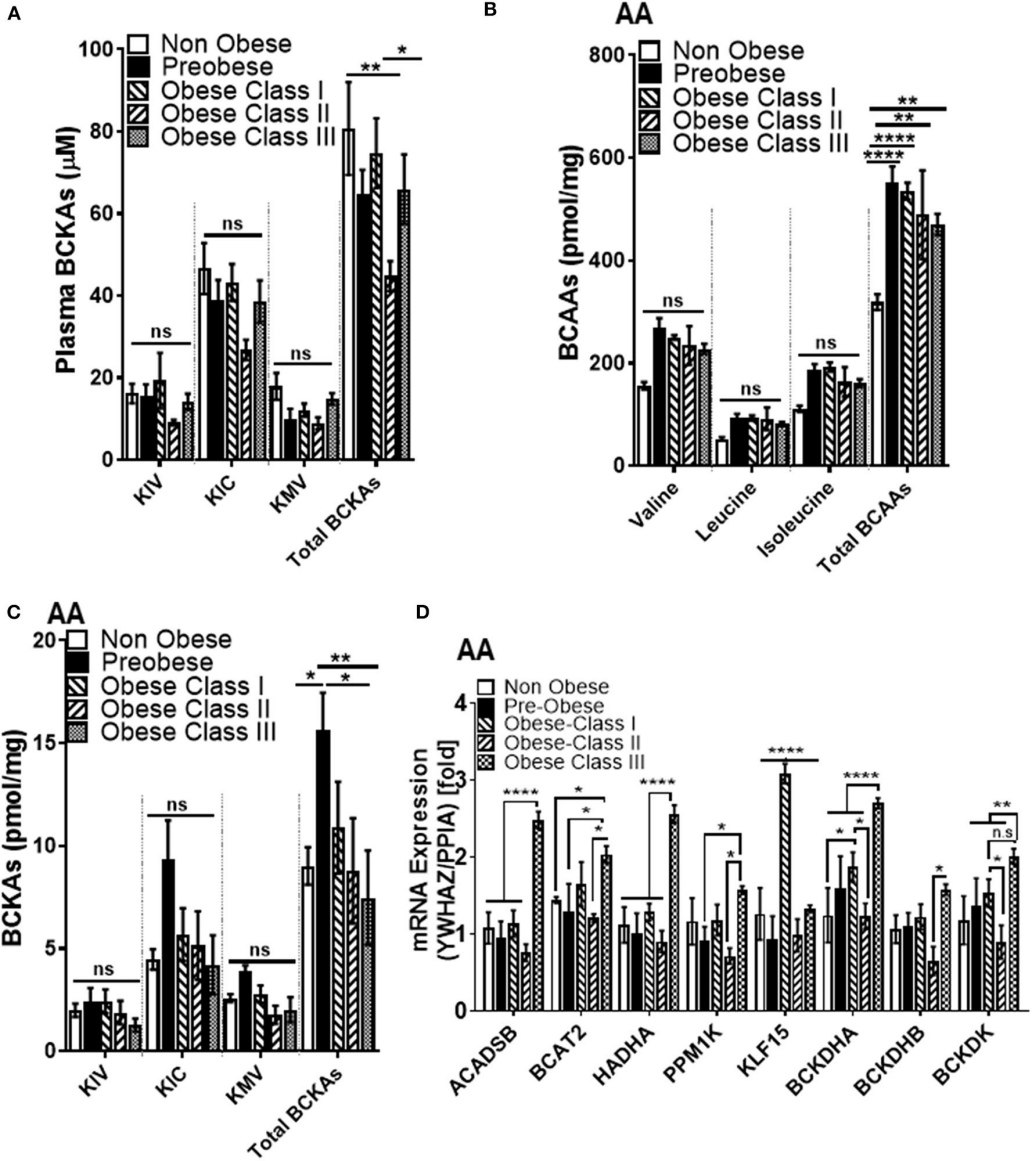
Intramyocellular BCAAs and BCAA catabolic enzyme mRNA expression are increased in the AA tissues of cardiac surgery patients with severe obesity. **(A)** Free plasma BCKA levels measured by UPLC MSMS in the non-obese (*n* = 9), pre-obese (*n* = 10) and class I obese (*n* = 10), class II obese (*n* = 8) and class III obese (*n* = 10) cardiac surgery patients. Intracellular **(B)** BCAAs and **(C)** BCKAs measured by UPLC MSMS in the AA of non-obese (*n* = 5), pre-obese (*n* = 5), class I obese (*n* = 5), class II obese (*n* = 5) and class III obese (*n* = 5) cardiac surgery patients. **(D)** Quantification of *ACADSB, BCAT2,HADHA, PPM1K, KLF15, BCKDHA, BCKDHB*, and *BCKDK* mRNA expression corrected to *YWHAZ* and *PPIA* levels (39) in the AA of non-obese (*n* = 6), pre-obese (*n* = 6) and obese class I (*n* = 6), class II (*n* = 6) and class III (*n* = 6) cardiac surgery patients. Statistical analysis was performed using a two-way ANOVA followed by a Tukey's multiple comparison test; *p < 0.05, **p < 0.01, ****p < 0.0001 as indicated (40).

We next wanted to investigate whether changes in BCAA catabolic enzyme expression in the SAT could influence systemic and AA BCAA catabolism in our patient cohort. We found that total BCKAs in the SAT trended to increase in the pre-obese and class I obese patients but remained unaltered in patients with class II and class III obesity (Supplementary Figure 2B). Total plasma BCKAs showed a decreasing trend with the severity of obesity, with class II obese patients showing the most pronounced change (Figure 4A). Intramyocellular BCKA levels are augmented in the failing hearts of mouse and human (33, 34). We therefore reasoned that the reduced systemic BCKA levels might signify an increased uptake and increased catabolism in the non-adipose depots, such as cardiac muscle and thus investigated the intracellular BCKA and BCAA levels in the AA from the cardiac surgery patients. Our UPLC MS data revealed that total intracellular BCAA levels were significantly increased with obesity in AA (Figure 4B). BCKAs were elevated in the AA of pre-obese patients while it was decreased in class II and III obese patients (when compared to the pre-obese patients) (Figure 4C). To ascertain whether changes in BCKAs were an outcome of altered expression of BCAA catabolic enzymes, the mRNA and protein expression of the BCAA catabolic enzymes was measured in AA. Increases in the mRNA levels of *ACADSB, BCAT2, HADHA* and both the *A* and *B* subunits of *BCKDH* suggested that BCAA catabolism is upregulated in the AA of obese cardiac surgery patients and particularly pronounced in class III obesity (Figure 4D). This observation was also supported by augmented *PPM1K* mRNA levels in class III obese patients (when compared to pre-obese patients) (Figure 4D). Increase in *KLF15* mRNA levels was observed in class I obese patients but it remained unaltered in class II and III obesity (Figure 4D). *BCKDK* mRNA expression, remained unaltered in the class I obese patients, decreased in class II obesity and increased in the class III obese group (when compared to the pre-obese patients) (Figure 4D). We observed a negative correlation between intramyocellular leucine (Supplementary Figure 3A), isoleucine (Supplementary Figure 3B) and total BCAAs (Supplementary Figure 3C) with *BCKDHB* mRNA levels in the AA. Increased *BCAT2* mRNA expression in the AA was also correlated with reduced KIV/Val ratio (Supplementary Figure 3D) suggesting an increased mRNA expression of BCAA metabolizing enzymes drives BCAA catabolism in the AA tissues of patients with obesity undergoing cardiac surgery.

**Figure 5:**
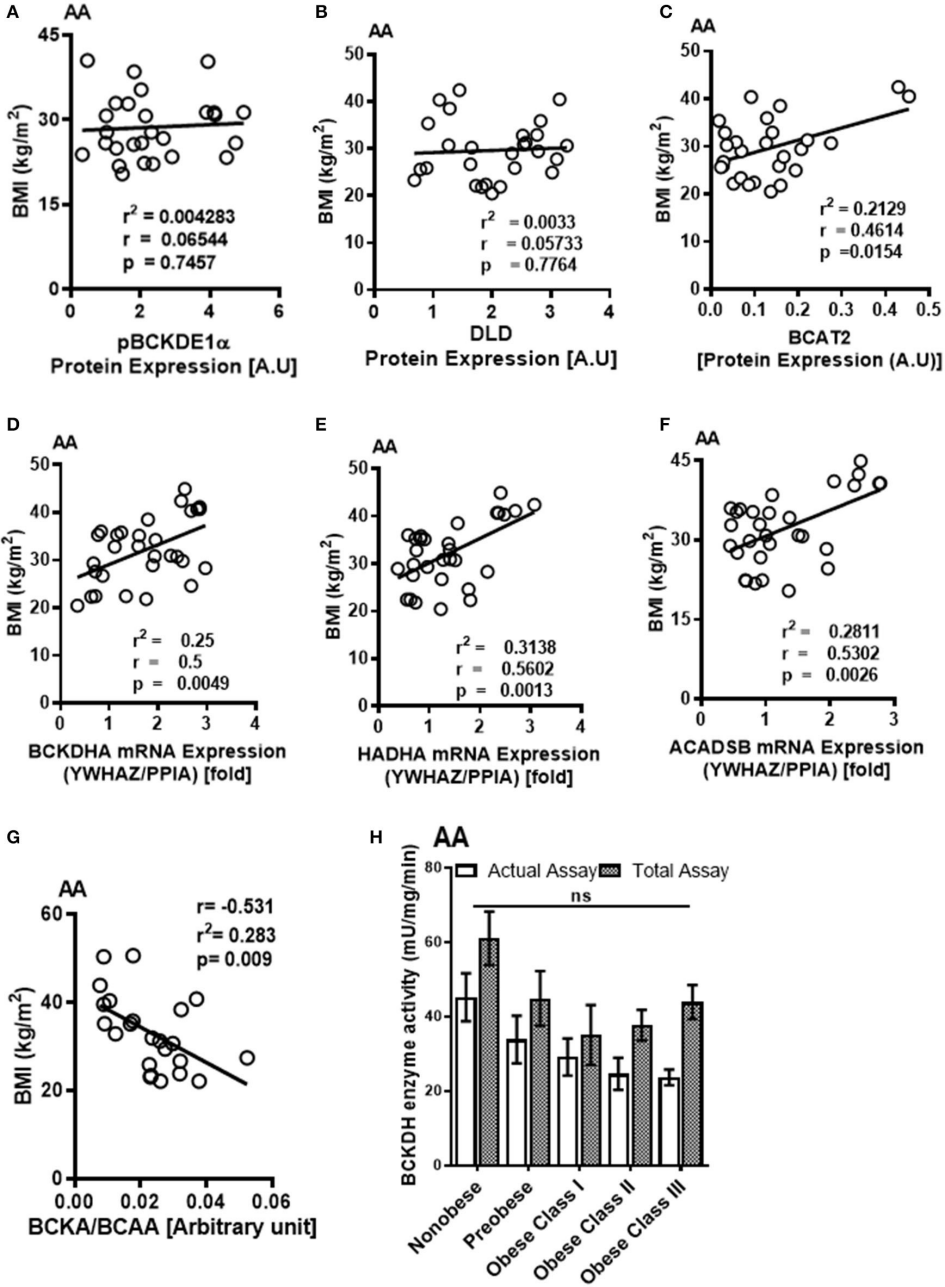
Increased mRNA expression of BCAA catabolic enzymes and altered BCKA to BCAA levels in the AA of cardiac surgery patients with obesity are associated with BMI. Linear regression of **(A)** pBCKDE1α, **(B)** DLD, and **(C)** BCAT2 protein expression in the AA tissues of non-obese (*n* = 7), pre-obese (*n* = 7), class I obese (*n* = 8) and class II obese (*n* = 6) cardiac surgery patients correlated with BMI. Linear regression of **(D)** *BCKDHA*, **(E)** *HADHA*, and **(F)** *ACADSB* mRNA expression in the AA tissues of non-obese (*n* = 6), pre-obese (*n* = 6), class I obese (*n* = 6), class II obese (*n* = 6) and class III obese (*n* = 6) cardiac surgery patients correlated with BMI. **(G)** Linear regression of the intramyocellular BCKA to BCAA ratio of non-obese (*n* = 5), pre-obese (*n* = 5), class I obese (*n* = 5), class II obese (*n* = 5) and class III obese (*n* = 5) cardiac surgery patients correlated with BMI. Statistical analysis was performed using one-way ANOVA; followed by a Tukey's multiple comparison test; *p* < 0.05 was considered significant. **(H)** Total and actual BCKDH enzyme activity corrected to protein levels, measured at Vmax (*t* = 15min) in the AA of non-obese (*n* = 8), pre-obese (*n* = 7), class I obese (*n* = 6), class II obese (*n* = 9) and class III obese (*n* = 8). Statistical analysis was performed using a two-way ANOVA followed by a Tukey's multiple comparison test; *p* < 0.05 was considered significant.

Although pBCKDE1 protein levels were increased in the AA tissues of obese patients (Supplementary Figure 4A), they did not correlate with BMI (Figure 5A). BCKDH protein levels were lower in the pre-obese group (Supplementary Figure 3A) but remained unaltered in the class I obese compared to non-obese patients. Moreover, DLD protein levels were unchanged between the non-obese and pre-obese patients (Supplementary Figure 4A) but decreased in the class I obese patients and did not correlate with BMI (Figure 5B). KLF15 protein levels remained unaltered in class I obese patients (Supplementary Figure 4A). Mitochondrial BCAT2 positively correlated with BMI (Figure 5C), signifying an increase in BCAA catabolism in the AA of obese patients despite downregulation of KLF15, in the AA tissues of obese patients with underlying cardiovascular defects. mRNA levels of *BCKDH, ACADSB* and *HADHA* positively correlated with BMI (Figures 5D–F), as well as with hip and waist circumference (Table 5). Since the mRNA expression of BCAA catabolizing enzymes did not always reflect in changes in protein content, we determined BCKDH enzyme activity in the AA tissues of obese cardiac surgery patients. Both the actual and total BCKDH activity in the AA remained unchanged across all obese groups (Figure 5H, Supplementary Figure 4B) suggesting that unlike SAT wherein the BCKDH enzyme activity decreased in the severely obese patients, the activity was maintained in the AA tissues.

Ratio of specific and total BCKAs to BCAAs in the AA trended to decrease (Supplementary Figure 3B) and correlated with BMI (Figure 5G), waist circumference as well as hip circumference (Table 6), suggesting that increased BCAA levels in the AA is associated with the severity of obesity.

Taken together, our data suggests that mRNA expression of BCAA metabolizing enzymes decreased in SAT and increased in AA in cardiac surgery patients with obesity. Within SAT, the decrease in mRNA expression of BCAA catabolizing enzymes corresponded with reduced BCKDH enzyme activity signifying inhibition of BCAA catabolic activity. Within AA, the increased expression of BCAA catabolizing enzymes was accompanied by increased intracellular BCAA levels and reduced BCKA levels without any decrease in BCKDH activity. Our findings agree with a recent *in vivo* isotope tracing study in mice demonstrating a shift in BCAA catabolism from adipose tissue toward muscle with IR progression (44).

### BCAA Catabolic Alterations in SAT and AA Correlates With Markers of Tissue Dysfunction and Influence Post-operative Outcomes

**Figure 6:**
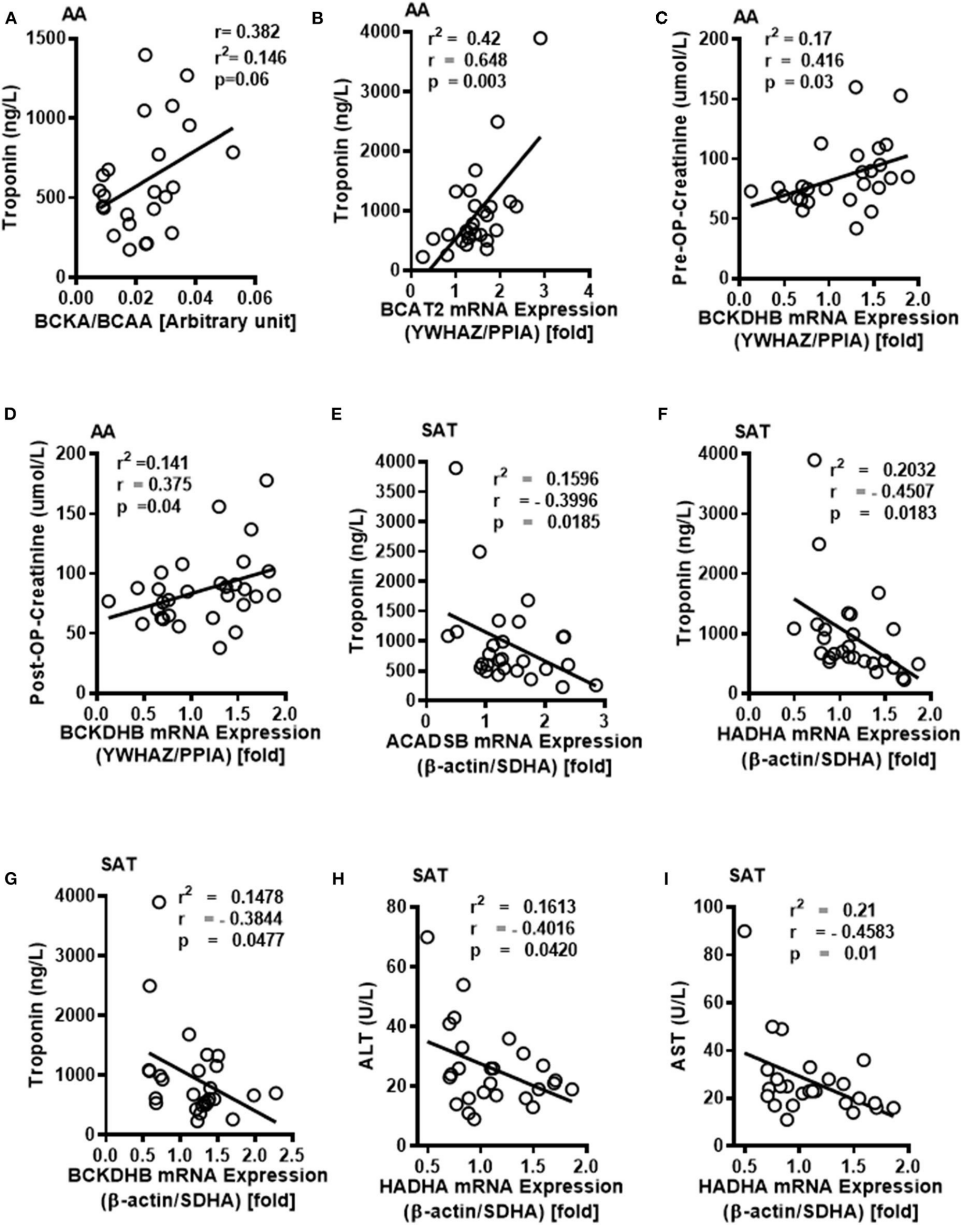
Intramyocellular BCAAs and altered BCAA catabolic enzyme expressions in the SAT and AA tissues are associated with markers of tissue dysfunction in cardiac surgery patients with increasing obesity. **(A)** Linear regression of BCKA to BCAA levels in the AA tissues of non-obese (*n* = 5), pre-obese (*n* = 5), class I obese (*n* = 5), class II obese (*n* = 5) and class III obese (*n* = 5) cardiac surgery patients correlated with pre- operative plasma troponin levels. In the AA tissues of non-obese (*n* = 6), pre-obese (*n* = 6), class I obese (*n* = 6), class II obese (*n* = 6) and class III obese (*n* = 6) patients undergoing cardiac surgery, linear regression of **(B)** BCAT2 mRNA expression were correlated with post-operative plasma troponin levels, **(C,D)** Linear regression of BCKDHB mRNA levels were correlated with pre and post-operative plasma creatinine levels. In the SAT of non-obese (*n* = 6), pre-obese (*n* = 6), class I obese (*n* = 6), class II obese (*n* = 6) and class III obese (*n* = 6) cardiac surgery patients, linear regression of **(E)** *ACADSB*, **(F)** *HADHA* and **(G)** *BCKDHB* mRNA expression correlated with post-surgery plasma troponin levels, **(H,I)** *HADHA* mRNA correlated with **(H)** plasma ALT and **(I)** AST levels. Statistical analysis was performed using one-way ANOVA; followed by a Tukey's multiple comparison test; *p* < 0.05 was considered significant.

To examine whether the altered BCAA flux in the heart and adipose induces hepatic toxicity and cardio-renal injury, we determined correlations between changes in BCAA catabolic enzyme expression and tissue damage markers in the cardiac surgery patients. We found a positive correlation of intramyocellular ratio of BCKAs to BCAAs (Figure 6A), *BCAT2* mRNA (Figure 6B) as well as ACADSB mRNA levels (Table 6) with post-operative troponin, a cardiac tissue damage marker. In our study, we found a strong negative correlation of HDL with increasing intramyocellular levels of all three individual BCAAs as well as total BCAAs (Table 6). In our study, *BCKDHB* mRNA expression in the AA tissue (Figures 6C,D) and intramyocellular valine (Table 6) positively correlated with both the pre as well as post-operative creatinine levels, a critical renal damage marker. A negative correlation was also established between the post-operative creatinine levels and the total and actual BCKDH activity (Table 6). Furthermore, mRNA levels of both the *A* and *B* subunits of *BCKDH* and *BCKDK* as well as protein levels of BCKDHA, BCAT2, and PPM1K were also positively correlated with blood urea levels (Tables 4, 5), suggesting that the impairment in kidney function post-surgery may be influenced by altered BCAA flux in the heart. Our data also revealed a negative correlation between HIBCH (3-hydroxyisobutyryl-CoA hydrolase), SOAT1 and ERAB/HADHII (endoplasmic-reticulum-associated amyloid beta-peptide-binding) protein expression and circulating glycine levels (Table 4), known to regulate lipid homeostasis and cholesterol transport (45). Increased mRNA expression of BCAA catabolic enzymes in the heart was also found to be detrimental for proper cardiac function, where *PPM1K* mRNA levels and HIBCH (3-hydroxyisobutyryl-CoA hydrolase) protein levels were negatively correlated with left ventricular end diastolic pressure (LVEDP) (Tables 4, 5). Furthermore, c- reactive protein (CRP) levels were positively correlated with mRNA expression of both the subunits of *BCKDH* as well as *BCKDK* in the AA tissues of the patients (Table 5). Decreased BCKAs and the corresponding increase in BCAA levels in the AA was also correlated with the severity of heart failure, based on the NYHA classification, which was also evident with increased length of stay of the patients following cardiac surgery (Table 6). Interestingly increased levels of HIBCH and SOAT1, protein involved in BCAA degradation significantly correlated with lower leucocyte counts and moreover lower leucocyte and neutrophil counts correlated significantly with increased PPM1K, BCKDK and DLD proteins (Table 3) questioning the likely role for BCAAs in the regulation of blood cell turnover.

Conversely in SAT, post-operative troponin was negatively correlated with the *ACASB, HADHA* and *BCKDHB* mRNA levels (Figures 6E–G). We found that increasing levels of pre-operative liver injury markers, alanine transaminase (ALT) and aspartate transaminase (AST), are negatively correlated with *HADHA* mRNA expression in SAT (Figures 6H,I). BCAA catabolic enzyme expression was also associated with cardiac function, where SOAT1 protein positively correlated with LVEDP (Table 2). Concomitant with the association of dysregulated BCAA catabolic enzymes in the adipose with cardiac injury, we also found a positive correlation between *BCKDHB* mRNA levels and fasting cholesterol and LDL levels (Table 3). Taken together, we show that BCAA catabolic enzyme dysregulation in the SAT and AA tissues of obese patients robustly associate with adverse cardiometabolic parameters in obese patients undergoing cardiac surgery.

**Figure 7:**
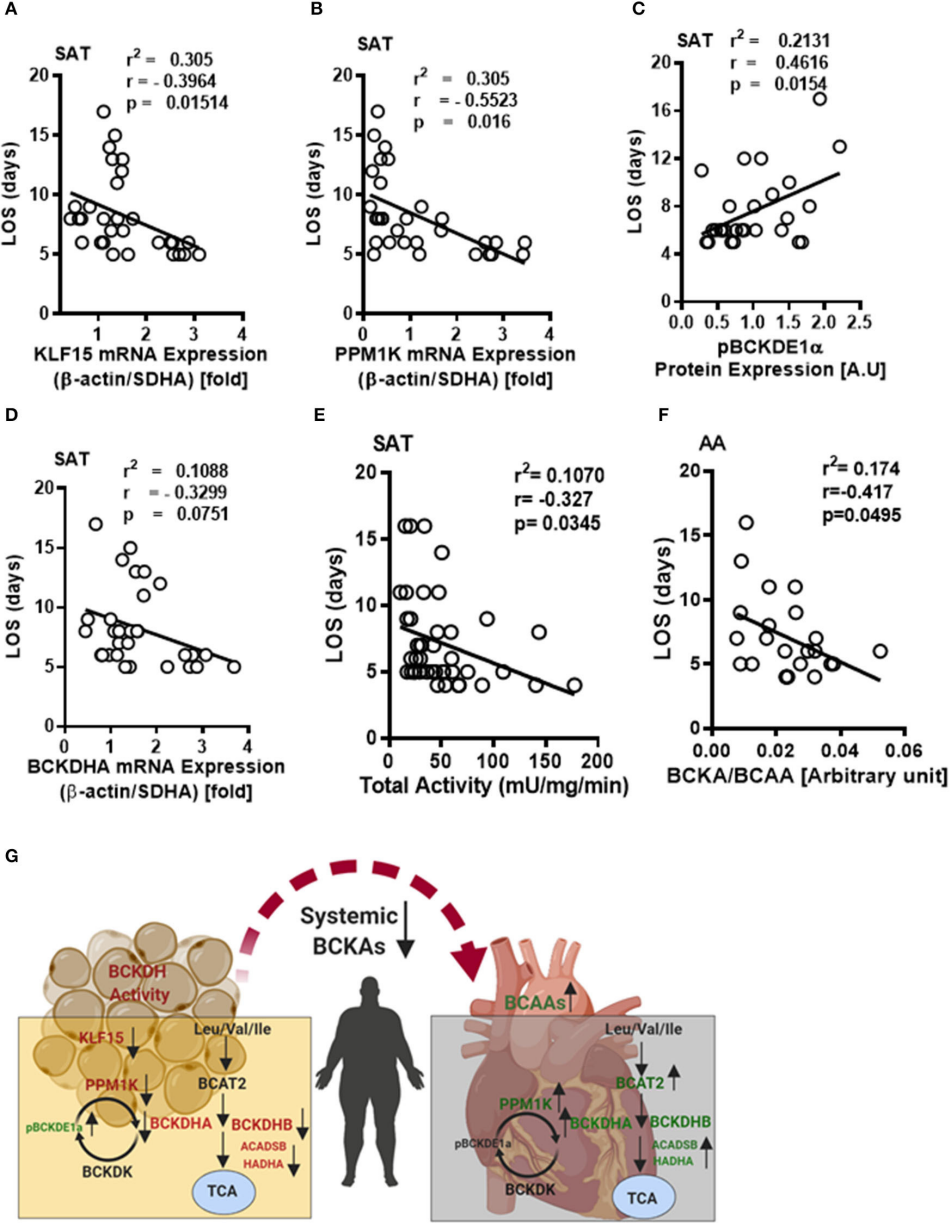
Decreased BCKDH enzyme activity, BCAA metabolic enzyme expressions in the SAT and altered intracellular BCAA levels in the AA are associated with length of stay following cardiac surgery in obese patients. Linear regression of **(A)** *KLF15*, **(B)** *PP1MK* mRNA levels, **(C)** pBCKDE1α, and **(D)** BCKDHA protein levels correlated with the length of stay in the hospital following surgery in the SAT of non-obese (*n* = 7), pre-obese (*n* = 7), class I obese (*n* = 8) and class II obese (*n* = 6) cardiac surgery patients. **(E)** Linear regression of total BCKDH activity at Vmax correlated with the duration of stay in the hospital following surgery in the SAT of non-obese (*n* = 7), pre-obese (*n* = 9), class I obese (*n* = 9), class II obese (*n* = 9) and class III obese (*n* = 8) patients undergoing cardiac surgery. **(F)** Linear regression of the ratio of intramyocellular BCKA to BCAA correlated with the length of stay following surgery in the AA of non-obese (*n* = 5), pre-obese (*n* = 5), class I obese (*n* = 5), class II obese (*n* = 5) and class III obese (*n* = 5) cardiac surgery patients. Statistical analysis was performed using one-way ANOVA; followed by a Tukey's multiple comparison test; *p* < 0.05 was considered significant. **(G)** Summary of the tissue specific alterations of BCAA catabolic enzyme expression, BCAA levels and BCKDH enzyme activity in the SAT and AA tissues of cardiac surgery patients with underlying obesity.

We next investigated whether the altered BCAA catabolic enzyme expression in SAT and AA tissues are indicative of post-operative outcomes in obese patients following cardiac surgery. The length of stay (LOS) in the hospital following surgery is an important determinant of post-operative outcomes. In our study, reduced BCAA catabolism in the SAT of obese patients was associated with increased LOS. mRNA levels of *KLF15* and *PPM1K* were inversely correlated with LOS (Figures 7A,B), while levels of phosphorylated BCKDH E1a subunit (pBCKDE1a) positively correlated with LOS (Figure 7C). *BCKDHA* mRNA expression trended to be negatively correlated with LOS (Figure 7D) while downregulation of both the actual (Table 6) and total BCKDH enzyme activity (Figure 7E) in the SAT was correlated with increased duration of hospital discharge. Moreover, the levels of the isoleucine derived BCKA, KMV (Table 6) as well as BCKA/BCAA ratio was negatively correlated with LOS in the AA (Figure 7F). Longer duration of stay also correlated with reduced levels of the ratio of KMV/ isoleucine and KIC/leucine (Table 6), Together, these findings suggest that dysregulated BCAA catabolism in the SAT and AA is related to a prolonged LOS post cardiac surgery and likely predisposes to a less favorable hospital discharge (Figure 7G).

## Discussion

Plasma BCAA levels have been widely proposed as biomarkers of cardio-metabolic diseases (14, 17, 35, 46). However, several prospective metabolome-wide studies of incident coronary heart disease (19, 20), coronary artery disease (21–23) and myocardial infarction (MI) (21) do not support the paradigm that elevated circulating BCAAs are causally associated with CVD. We performed this study to determine if BCAA metabolizing enzymes and intracellular BCAA content are more definitive predictors of cardiometabolic health. For the first time in obese patients undergoing cardiac surgery, our study demonstrated a co-ordinate regulation of BCAA catabolic enzymes in the heart and subcutaneous adipose tissue. Moreover, our data indicate that altered myocellular BCAA content as well as BCAA metabolic enzyme expression in obese heart and adipose tissue correlated with worsening adverse outcomes following cardiac surgery.

Prior studies have reported the importance of white adipose tissue in modulating whole body BCAA metabolism. Indeed, downregulation of BCATm and BCKDH in the adipose tissue has been associated with increased levels of plasma BCAA in both obese humans (7, 47, 48) and genetic or diet-induced obesity models of rodents (49–51). In our study, we procured the SAT surrounding the heart of patients undergoing cardiac surgery. Interestingly, our data showed that in early stages of obesity (pre-obese), BCAA catabolizing enzyme expressions was upregulated in the SAT when compared to the non-obese patients. However, in the SAT of patients with class II and class III obesity, mRNA and protein levels of BCAA catabolic enzymes were markedly downregulated along with increased levels of pBCKDE1α and BCKDK. Consistently SAT BCKDH activity was also downregulated suggesting disrupted BCAA catabolism in the with severe obesity in a setting of underlying heart disease. Correlation analysis of the BCAA catabolizing enzymes and BCKDH enzyme activity with BMI as well as indicators of IR demonstrated robust association in the SAT. Our findings in the human obese cardiac surgery patients is supported by a prior study by Lackey et al., demonstrating significantly reduced mRNA levels of BCKDH subunits in the SAT of Pima Indian population with obesity (52). Contrary to rodent studies demonstrating an increase in circulating BCAAs due to downregulation of adipose BCAA catabolic enzymes (41), BCAA catabolizing enzyme expression remodeling in SAT of obese cardiac surgery patients in our study did not reflect in changes in plasma BCAA levels. Indeed, Patterson et al. demonstrated that a relatively lower rate of leucine release from abdominal subcutaneous adipose tissue of obese women than in the lean women (53), suggesting that adipose tissue acts as a sink of circulating BCAAs. Therefore, it is plausible that decreased levels of BCAT in the SAT is inadequate to transaminate BCAAs, which coupled with reduced BCAA catabolic enzyme expression in the SAT might not trigger changes in intracellular BCKA levels, as observed in our study.

Tissue specific regulation of BCAA catabolic fate are reported in murine models of IR (41, 54, 55). Reduction in systemic BCKA levels in our obese patient cohort prompted us to investigate BCAA metabolism in the heart. Isotope labeling studies have demonstrated the increase and decrease in BCAA oxidative flux in the skeletal muscle and adipose, respectively, during the progression of IR in obese mouse models (44) resulting in elevated acyl carnitine accumulation (54). In our study, intramyocellular BCAAs were augmented in the pre-obese and obese cardiac surgery patients, while the BCKAs were only increased in the AA of pre-obese patients. Contrary to previous reports in human ventricular appendage from end stage failing hearts (33) and explanted heart samples from patients with diabetic cardiomyopathy (34), mRNA expression of BCAA metabolism enzymes in AA were upregulated in the severely obese patients in our study. The BCAA catabolic enzyme alterations observed at the mRNA levels were independent of changes in their protein expression or BCKDH activity, suggesting a probability of posttranslational regulation. Correlation analysis of the mRNA expression of the BCAA catabolizing enzymes in the AA demonstrated association with BMI. The different observations in our patient cohort as opposed to prior reports could be attributed to the type of cardiac tissue procured, stage of heart failure, and presence of underlying co-morbidities as well as different treatment modalities the patients are subjected to pre- and post-surgery.

Moreover, reduced BCKA levels as well as BCKA/BCAA ratio in the AA was associated with different parameters of obesity suggesting that increased accumulation of BCAAs in the AA of cardiac surgery patients with obesity precipitates obesity related co-morbidities.

Perturbations of BCAA catabolizing enzymes were reported to be associated with altered expression of the heart failure markers, atrial natriuretic factor (ANF), beta myosin heavy chain (β-MHC) (33). Our study examined whether tissue specific alterations of BCAA flux and changes in enzyme expression in the AA and SAT correlated with the prognosis and post surgical outcomes of obese patients undergoing cardiac surgery. Elevated levels of troponin and creatinine are reported signatures of tissue damage following cardiac surgery (56–58) which significantly affects the postoperative outcomes in terms of better prognosis as well as discharge times. Some of the common complications following cardiac surgery are the development of acute kidney injury (59) and increases in transient post-operative liver function tests (60). Alternatively, pre-operative chronic kidney disease (61) and liver dysfunction (62) are reported to worsen cardiac surgery outcomes (63). Our data demonstrated that dysregulated BCAA catabolism in the AA is correlated with the cardiac and renal damage markers levels, specifically, post-operative troponin, creatinine, blood urea and CRP reflecting poorer post-surgical outcomes likely due to the toxicity of downstream BCAA metabolites that are not necessarily BCKAs, in the myocyte, liver and kidneys. Dysregulated BCAA catabolic enzymes in the AA of obese patients were also associated with an increased propensity for cardiac failure as indicated by reduction in the LVEDP values. Reduced HDL-cholesterol levels are associated with incidence of heart diseases (64) and our findings reflected that increased BCAA levels in the AA are correlated with marked reduction in HDL in our patient cohort. Prior reports have shown that levels of plasma dimethyl glycine and glycine improves risk prediction in patients with coronary heart disease and MI (45, 65) and increased plasma glycine levels are protective against the risk of developing CVD (45, 66). Our findings suggest that increases in BCAA catabolic enzyme expression in the AA alters plasma glycine profile making the heart more susceptible to cardiac injury. Altered BCAA catabolism in the SAT was also correlated with post-operative troponin, pre-operative hepatic damage markers, AST, ALT, fasting cholesterol, LDL levels as well as markers of insulin resistance. Decreased BCAA catabolic enzyme expression in the SAT was also correlated with increased LVEDP values and longer discharge times following surgery. Moreover, cardiomyopathic human hearts exhibit downregulation of BCAA catabolizing enzyme gene expression with concomitant upregulation of intramyocardial BCKAs signifying that defective BCAA catabolism is a metabolic phenotype of CVDs (33). In addition, systemic BCAA levels and NT-pro-B-type natriuretic peptide (NT-proBNP) yielded a stronger prognostic value and robustly predicted adverse cardiovascular outcomes in patients with ST-segment elevation myocardial infarction (STEMI) and acute heart failure (AHF) when compared to NT-proBNP alone (17). Our findings thus highlight the importance of adequate tissue specific regulation of BCAA catabolism, the imbalance of which could result in metabolic inflexibility that may precipitate cardio-renal-hepatic injury following cardiac surgery in a setting of obesity. Our study is in agreement with prior reports demonstrating tissue compartment specific regulation and inter tissue cross-talk of BCAA metabolizing enzymes and catabolic intermediates (52, 54, 67).

## Limitations of the Study

Since this study was solely conducted in humans, we have highlighted specific limitations of this study. All participants in this study were cardiac surgery patients stratified according to their BMIs. To examine if plasma BCAA levels associate with obesity and its related co-morbidities, non-obese patients were chosen as controls. Despite control patients having a healthy BMI, they are undeniably patients, with underlying chronic cardiac complications, on specific treatment regimen pre- and post-surgery. The issue of gender differences on cardiometabolic functional outcome of patients can influence our interpretation of the presented data. Notably, due to minimal tissue availability our participant numbers are variable across different experiments, limiting our ability to examine 3-HIB, BAIBA and acyl carnitine content in addition to BCAA and BCKA levels, which might influence intramyocellular BCAA and BCKA flux. Furthermore, in the presented data correlations are unadjusted and have been established based on *p*-value significance. Lastly the hetero-cellularity of the heart and the cell specific contributions of BCAAs and BCKAs in our data interpretation is paramount for predicting CVD outcomes.

## Conclusion

Taken together, our clinical data suggests that systemic BCAA levels *per se* is not adequate to explain the underlying molecular complexities in the setting of CVD precipitated by obesity. We propose that interpreting the pathobiology of BCAA dysmetabolism requires looking beyond systemic BCAA levels and more so toward tissue specific alterations in the expression of BCAA metabolizing enzymes and levels of intracellular BCAAs and BCKAs. We have included different stratifications of obesity in our study which was important to address as our results indicate differences between the pre-obese and class I-III obese groups. An important observation in this study was that in obese patients undergoing cardiac surgery intracellular BCAAs and BCAA catabolic enzyme expression, but not systemic BCAAs, correlated with BMI. We infer that dysregulation of BCAA catabolizing enzymes mRNA expression is differentially regulated in multiple tissue depots highlighting their compartment specific contribution in driving organ metabolism and function (Figure 7G). Moreover, poorer cardiac health and surgical complications following surgery corresponded with dysregulation of BCAA catabolic enzyme expression at the level of mRNA, protein, enzyme activity and metabolite (BCAA/BCKA) content in the myocardium. This study proposes that in a setting of obesity, dysregulated BCAA catabolism could be an effective surrogate to determine cardiac surgery outcomes and plausibly predict premature re-hospitalization.

## Experimental Methods

### Clinical Sampling

Human tissue samples were collected from patients undergoing elective, first-time cardiac surgery (coronary artery bypass graft and/or valve surgery) at the New Brunswick Heart Centre in Saint John, NB, Canada who provided consent to be enrolled, as part of a previously described study (40), which was part of the OPOS trial (Trial registration NCT03248921) (68). All patients met the inclusion criteria and did not fulfill any exclusion criteria, as described previously (40). Our study cohort included 17 non obese (M = 11, F = 6), 19 pre-obese (M = 19, F = 0), 14 obese class I (M = 11, F = 3), 17 obese class II (M = 14, F = 3) and 12 obese class II (M = 9, F = 3) patients.

### Sample Collection and Anthropometric Measurements

Immediately before surgery, a venous blood sample was collected and plasma was separated and stored at −80°C for long term storage. During the surgery, samples of the right atrial appendage (AA) and thoracic subcutaneous adipose tissue (SAT) were excised, frozen in liquid nitrogen, and stored at −80°C until further processing for quantitative polymerase chain reaction (qPCR) and immunoblot analysis, as described previously (40). Anthropometric measurements were done during patient enrolment for surgery by the Cardiac Surgery Research Team at Saint John Regional Hospital (SJRH). Weight, height, waist circumference and hip circumference were measured.

### Biochemical Measurements

Measurement of ALT, AST, creatinine, glucose, insulin, troponin T and HOMA-IR levels in plasma samples were conducted by the Department of Laboratory Medicine, Saint John Regional Hospital (SJRH). Clinical data from patients who participated in the study was retrieved by the Cardiac Surgery Research Team at SJRH. This included information regarding demographics (BMI, age, sex), blood cell type, quantity and morphology (hemoglobin, hematocrit, platelets, leukocytes, erythrocytes, neutrophils, lymphocytes), electrolytes (sodium, potassium, chloride), blood lipids (cholesterol, triglycerides, fasting HDL cholesterol, fasting LDL cholesterol, fasting non HDL cholesterol), glucose tolerance (HbA1C, random glucose), cardiac function (pre and post-operative troponin, ejection fraction, LVEDP, NYHA classification), post-operative outcomes (length of stay), and co-morbidities (smoking, diabetes, hypertension, creatinine, urea (blood), renal failure). All studies were approved by the Research Ethics Board of the Saint John Regional Hospital, NB, Canada (protocol #2014–2006). A written informed consent was obtained from all participants, and all experiments were performed in accordance with relevant guidelines and regulations.

### Tissue Processing, Subcellular Fractionation, and Immunoblotting

15–20 mg of human subcutaneous adipose tissue and atrial appendage were powdered and homogenized using a tissue homogenizer (Omni TH, Omni International) in ice-cold lysis buffer [containing 20 mM Tris-HCl, pH 7.4, 5 mM EDTA, 10 mM Na_4_P_2_O7 (567540; Calbiochem, NJ, USA), 100 mM NaF, 1% Nonidet P-40, 2 mM Na_3_VO_4_, protease inhibitor (P8340, 10 μl/ml; Sigma, MO, USA) and phosphatase inhibitor (524628, 10 μl/ml, Calbiochem, NJ, USA)]. BCA protein assay kit (23255; Pierce, Thermo Fisher Scientific, MA, USA) was used to determine the protein concentrations. 30–35 μg of protein was subjected to SDS-PAGE and then transferred onto nitrocellulose membranes. Proteins were visualized using a reversible Coomassie stain (24580, Pierce, Thermo Fisher Scientific, MA, USA) and membranes were incubated with the primary antibodies which are provided in Table 7. Immunoblots were developed using the Western Lightning Plus-ECL enhanced chemiluminescence substrate (NEL103E001EA, Perkin Elmer, ON, Canada). Image lab software (Bio-Rad, CA, USA) was used to perform densitometric analysis. The brightness and contrast of the blots was uniformly adjusted, and images were cropped using Image lab software or Microsoft PowerPoint picture tools.

**Table 7:** Table of key resources.

**Reagent or resource**	**Source**	**Identifier**
**Antibodies**		
BCKDHA	My Biosource	MBS 275832
pBCKDE1α	Bethyl Lab	A303-567A
KLF15	Novus Biologicals	NBP2-24635
DLD	Santa Cruz Biotechnology Inc.	G-2; sc-365977
SOAT1	Santa Cruz Biotechnology Inc.	ACAT-1; sc-69836
BCKDK	My Biosource	MBS 275719
BCAT2	Cell Signaling Technologies	CST 9432
PPM1K	GeneTex	GTX 105934
ERAB	Santa Cruz Biotechnology Inc.	23; sc-136326
HIBCH	Santa Cruz Biotechnology Inc.	E-11; sc-515355
BCKDHB	Santa Cruz Biotechnology Inc.	H-6; sc-374630
Secondary antibodies	Santa Cruz Biotechnology Inc	sc-516102; sc-2357
**Chemicals**		
Leucine- d3	CDN Isotopes	D-1973
**Softwares**		
Prism 7	GraphPad	N/A
Image Lab 5.0	BioRad	N/A
SAS/STAT	SAS	N/A
qBase+	Biogazelle	N/A

*Fine chemicals if not noted otherwise are from Sigma*.

### qPCR Analysis

mRNA levels of BCAA metabolizing enzyme related genes in SAT and AA tissues were measured by quantitative PCR by employing optimal reference gene pairs which was validated as previously described (39). Primer information used for the study are provided in Table 8. Powdered tissue samples were homogenized in Ribozol (N580-CA, Amresco, OH, USA). RNA was isolated as per the manufacturer's instructions and QIAxcel Advanced System (Qiagen, Toronto, ON) was used to determine the RNA quality and quantity. One microgram of RNA of was used to synthesize cDNA using qScript cDNA supermix (CA101414-104, Quanta Biosciences). qPCR analysis was performed using PerfeCTa SYBR green Supermix Low ROX (Quanta Biosciences, MA, USA) and a ViiA7 real-time PCR machine (Thermo Fisher Scientific, CA, USA) as detailed previously (39). qBase + software (Biogazelle) was used to quantify mRNA expression (39).

**Table 8:** Details of specific primers and targets used in real-time qPCR experiments.

**Primer**	**Gene name /chromosome location**	**Sequence 5′-3′**	**Product size (bp)**	**Tm (°C)**	**GenBank ID**
h-BCAT2-F^a^	Branched chain amino acid transaminase 2/ 19q13.33	CGCTCCTGTTCGTCATTCTCT	134	59.1	NM_001190.4
h-BCAT2-R^a^		CCCACCTAACTTGTAGTTGCC		57.7	
h-PPM1K-F^a^	Protein phosphatase Mg2+/Mn2+ dependent 1K/ 4q22.1	ATAACCGCATTGATGAGCCAA	90	57.5	NM_152542
h- PPM1K-R^a^		CGCACCCCACATTTTCCAAG		59.2	
h-ACADSB-F^a^	acyl-CoA dehydrogenase short branched chain/ 10q26.13	GATGGCAAATGTAGACCCTACC	76	57.6	NM_001609
h-ACADSB-R^a^		AAGGCCCGGAGTATCACGA		60.0	
h-HADHA-F^a^	hydroxyacyl-CoA dehydrogenase trifunctional multienzyme complex subunit alpha/ 2p23.3	CTGCCCAAAATGGTGGGTGT	134	61.1	NM_000182
h-HADHA-R^a^		GGAGGTTTTAGTCCTGGTCCC		59.7	
h-KLF15 -F^b^	KLF15 Kruppel like factor 15/ 3q21.3	CGGCTGGAGGTTCTCGCGCTCTG	199	69.56	NM_014079
h- KLF15 -R^b^		AGGCTGGGGTTCAGGGCGCTTTC		69.45	
h-BCKDHA-F^a^	branched chain keto acid dehydrogenase E1 alpha polypeptide/ 19q13.2	CTACAAGAGCATGACACTGCTT	102	58.7	NM_000709
h-BCKDHA-R^a^		CCCTCCTCACCATAGTTGGTC		59.5	
h- BCKDHB-F^a^	BCKDHB branched chain keto acid dehydrogenase E1 subunit beta/ 6q14.1	TGGAGTCTTTAGATGCACTGTTG	109	57.5	NM_183050
h- BCKDHB-R^a^		CGCAATTCCGATTCCAAATCCAA		59.2	
h- BCKDHK-F^a^	BCKDK branched chain ketoacid dehydrogenase kinase/ 16p11.2	GACTTCCCTCCGATCAAGGAC	116	59.0	NM_005881
h- BCKDHK-R^a^		CTCTCACGTAGGCCCTCTG		58.2	

a *Primers reported by Jang et al. (69)*.

b *Primers were designed in the current study*.

### BCKDH Enzyme Activity Measurements

BCKDH activity in the AA and SAT were measured as described previously (70). Briefly, 60–70 mg of powdered AA and 75–80 mg of powdered SAT samples were homogenized in 300 μl of ice cold extraction buffer (50 mM HEPES, 3% Triton, 2 mM EDTA, 5 mM DTT, 0.5 mM thiamine pyrophosphate (TPP), 1 mM α-chloroisocaproate, 50 mM potassium fluoride, 2% bovine serum, 0.1 mM N-tosyl-L-phenylalanine choloromethyl ketone (TPCK), 0.1 mg/mL trypsin inhibitor, 0.02 mg/mL leupeptin (pH 7.4 at 4°C). Insoluble materials were pelleted by centrifugation at 21,200 g for 10 min at 4°C and the supernatant was removed carefully and transferred. Following tissue extraction, the supernatant was divided into two parts; one for BCKDH activity assay and one for protein estimation. For complete precipitation of the proteins, 27% cold poly-ethylene glycol 6000 (PEG-6000) was added to the supernatant (final concentration of PEG-6000 was 9%) and incubated for 20 min on ice. Precipitated proteins were pelleted by centrifugation at 21,200 g for 10 min at 4°C and supernatant was completely removed using vacuum. Precipitated proteins can be frozen at this point at −80°C for assay within 72 h. Precipitated proteins were resuspended in 60 μL of suspension buffer (25 mM HEPES, 0.1% Triton X-100, 0.2 mM EDTA, 0.4 mM TPP, 1 mM DTT, 50 mM potassium chloride and 0.02 mg/mL leupeptin, pH 7.4). Following resuspension of the precipitate, it is divided into two parts; for the actual BCKDH assay and for total BCKDH.

#### Measurement of Actual BCKDH Activity

Twenty microliter of suspension buffer was added to 20 μl of the resuspended precipitate; which was then mixed with the assay buffer (60 mM potassium phosphate, 4 mM MgCl_2_, 0.8 mM TPP, 0.8 mM CoA, 2 mM NAD, 0.2% Triton X-100, 4 mM DTT and 10 U/mL pig heart dihydrolipoamide dehydrogenase (E3). E3 and MgCl_2_ was added to the assay mixture just prior to the assay and pre-warmed to 30°C. Absorbance was recorded at 340 nm for 15 min at 37°C using Synergy H4 hybrid multimode plate reader (Biotek) to establish a baseline and reaction was initiated with 50 mM alpha ketoisovalerate (final 1 mM) and kinetic measurement was performed for 30 min.

#### Measurement of Total BCKDH Activity

Twenty microliter of resuspended precipitate was mixed with 20 μl of suspension buffer containing 2 mM MnCl_2_ and 2,000 U/mL of lambda phosphatase. This was further mixed with the assay buffer and the reaction mixture was incubated for 30 min at 37°C (for dephosphorylation of BCKDH). Following incubation, the reaction mixture was centrifuged at 8,000 g for 3 min to remove insoluble particles. Absorbance to establish the baseline was measured for 15 min, following which the reaction was initiated with the substrate (50 mM α-ketoisovalerate (final 1 mM) and kinetic measurement was done at 340 nm for 30 min.

### Plasma and Tissue BCAA and BCKA Measurements

#### Plasma/Tissue (BCAA and BCKA Extraction)

Twenty microliter of plasma sample, or 40–50 mg of powdered atrial appendage tissue or 50–70 mg of powdered subcutaneous adipose tissue 120 μl of internal standard (ISTD; 4 μg/ml in H_2_O) containing leucine-d3 (CDN Isotopes, D-1973), 40 μl of MilliQ water, 60 μl of 4 M perchloric acid (VWR, CA71007-908) were combined and vortexed. Proteins were precipitated via a 2 min sonication followed by ice bath for 10 min, twice and centrifuged (13,000 RPM, 15 min, 4°C) and the protein pellet was rinsed with 60 μl of 1 M perchloric acid, vortexed and protein precipitated as described above. The new supernatant was combined with the first portion. The sample was split into two 150 μl portions for BCAAs and BCKAs. For BCAAs, 150 μl of extract was neutralized with 60–75 μl of 2 M KOH (VWR, CABH9262-500G) to a pH of 6–10, vortexed and centrifuged (13,000 RPM, 5min, 4°C). The supernatant was transferred to a new tube. For the plasma samples, the precipitate was rinsed with 100 μl of MilliQ water (18Ω), vortexed and centrifuged (13,000 RPM, 5min, 4°C). Supernatants were frozen, freeze dried and reconstituted in 60 μl of 50:50 Methanol (MeOH) water (VWR, CAMX0486-6) to yield 4 μg/ml of internal standard. Samples were derivatized as per the BCAA and BCKA derivatization protocol below as per a prior study (71–74).

#### BCAA Derivatization and Quantification

Ten microliter of reconstituted extract was transferred to an autosampler vial and was combined with 70 μL of Borate Buffer (Waters, 186003836) from Waters AccQ-Tag Derivatization Kit (target pH: 8–10) and vortexed. Twenty microliter of AccQ-Tag Derivatization Agent (Waters, 186003836) was added, vortexed and let stand for 1 min. Samples were derivatized (55°C, 10 min) and vortexed. Derivatized samples were quantified with a Waters Acquity UPLC, Xevo-μ Tandem Mass Spectrometer and an AccQ-Tag Ultra RP Column 130 Å, 1.7 μm, 2.1 mm, 100 mm column using multiple reaction monitoring (MRM) and internal standard calibration (72, 73).

#### BCKA Derivatization and Quantification

25–50 μL of extract was combined with 50 μL of BCKA internal standard; 0.8 ng/μL of KIVd_7_ (made from sodium-2-Keto-3-methyl-d3-butyrate-3,4,4,4d4-0.5g; CDN Isotopes, D-6855) and 500 μL of 25 mM OPD in 2 M HCl (made from o-Phenylenediamine, 98%; VWR, CAAAA11946-30). The mixture was vortexed and then incubated at 80°C for 20 min with shaking and then cooled on ice for 5 min. The derivatized extract was centrifuged at 500 g for 15 min. The extract was transferred to a tube containing approximately 0.08 g sodium sulfate (VWR, CA71008-804) then 500 μl of ethyl acetate (ethyl acetate; VWR, CABDH83621.100) was added. The sample was vortexed, centrifuged at 500 g at room temperature for 15 min. The supernatant was transferred to a second tube containing 0.08 g of sodium sulfate. A second 500 μl of ethyl acetate was added to the first sodium sulfate tube, vortexed, and centrifuged as above. The supernatant was combined with the first portion in the second sodium sulfate tube. The combined sample was vortexed and centrifuged at 500 g for 15 min. The new supernatant was transferred to a new empty tube. Samples were vacuum centrifuged at 30°C for 45 min. Samples were reconstituted in 50 μL of 200 mM ammonium acetate (made from; ammonium Acetate, 98%; VWR, CA97061-014) and transferred to amber glass UPLC vials (Waters, 186001130C). BCKAs were quantified with a Waters Acquity UPLC, Xevo-μTandem Mass Spectrometer and an Acquity UPLC BEH C18 1.7 μm, 2.1 50 mm (Waters, 186004660) and ACQBEHC18 VanGuard 130 Å,1.7 μm, 2.1 × 5 mm (Waters, 186003975) using multiple reaction monitoring (MRM) and internal standard calibration as per a prior study (71, 74).

### Statistical Analysis

Data are expressed as mean ± standard error of the mean (SEM) unless otherwise indicated. Statistical and Spearman's correlation analyses were conducted using Prism software (GraphPad, CA, USA) and SAS statistical software version 9.4 (Toronto, ON), respectively. Comparisons between multiple groups were performed using two-way analysis of variance followed by a Tukey *post-hoc* test or one-way ANOVA followed by a Tukey *post-hoc* test or a Chi square goodness of fit test, as appropriate. Clinical parameters were plotted against tissue biomarkers and linear regression statistical analysis as well as Spearman correlations for normally and non-normally distributed variables was performed to assess for significance. All correlations are unadjusted. *P*-values of <0.05 were considered statistically significant.

## Data Availability Statement

All relevant data are within the paper and Supplementary Figures. The UPLC raw files generated for this study are available on request to the corresponding author.

## Ethics Statement

The studies involving human participants were reviewed and approved by Research Ethics Board of the Saint John Regional Hospital, NB, Canada (protocol #2014–2006). The patients/participants provided their written informed consent to participate in this study.

## Author Contributions

TP and DB designed the research. DB, KT, AB, and IH performed the experiments. DB, KD, and AM generated UPLC-MS data. LP and DB generated qPCR data. DB and TP analyzed and interpreted the data and wrote the paper. AY generated clinical statistics data. JS generated clinical diagnostics data. CA, HM, KB, JL, and AH assisted with clinical sample collection and provided intellectual inputs to clinical study. PK provided intellectual inputs and technical assistance. All authors contributed to the article and approved the submitted version.

## Conflict of Interest

The authors declare that the research was conducted in the absence of any commercial or financial relationships that could be construed as a potential conflict of interest.

## Abbreviations

AA: atrial appendage
ACADSB: short/branched-chain acyl CoA dehydrogenase
ACAT/SOAT1: acetyl CoA acetyl transferase-1
AHF: acute heart failure
BAIBA: β-aminoisobutyric acid
BCAA: branched-chain amino acid
BCKA: branched-chain keto acids
BCAT2: mitochondrial branched-chain acyl transferase
BCKDH: branched-chain ketoacid dehydrogenase
BCKDK: branched-chain ketoacid dehydrogenase kinase
BMI: body mass index
CABG: Coronary Artery Bypass Graft
CRP: c-reactive protein
CVD: cardiovascular diseases
DBT: dihydrolipoamide branched- chain transacylase E2
DLD: dihydrolipoamide dehydrogenase
ERAB: endoplasmic-reticulum-associated amyloid beta-peptide-binding protein
HADHA: hydroxyacyl CoA dehydrogenase alpha subunit
3-HIB: 3-hydroxyisobutyrate
HIBCH: 3-hydroxyisobutyryl-CoA hydrolase
HMGCS: hydroxymethyl glutaryl-CoA synthase
IVD: isovaleryl-CoA dehydrogenase
KLF15: kruppel-like factor-15
LPL: lipoprotein lipase
LVEDP: left ventricular end diastolic pressure
NT-proBNP: NT-pro-B-type natriuretic peptide
OXCT: 3-oxoacid CoA-transferase
PCCB: propionyl-CoA carboxylase
PPM1K: protein phosphatase Mg2+/Mn2+phosphatase
ROS: reactive oxygen species
SAT: subcutaneous adipose tissue
STEMI: ST-segment elevation myocardial infarction
T2DM: type 2 diabetes mellitus
UPLC-MSMS: ultraperformance liquid chromatography-tandem mass spectrometry.
