# Supplementary material for: Adverse Outcomes in Obese Cardiac Surgery Patients Correlates With Altered Branched-Chain Amino Acid Catabolism in Adipose Tissue and Heart
Source: Front Endocrinol (Lausanne). 2020 Aug 7;11:534. doi: 10.3389/fendo.2020.00534 (PMC7438793; doi:10.3389/fendo.2020.00534)
Supplement: Supplementary file 1 [file Data_Sheet_1.pdf]

Figure S1

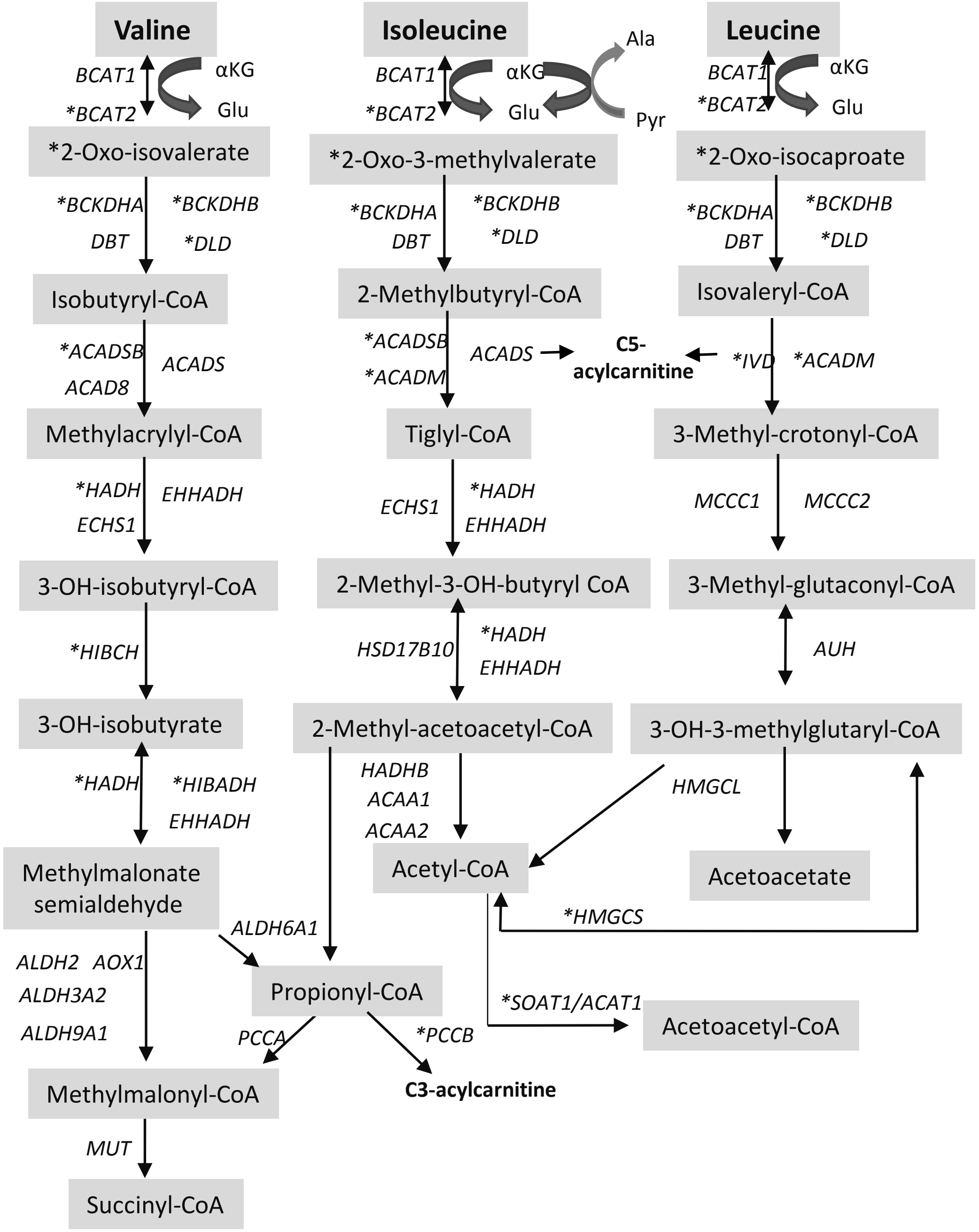

Figure S1: Summary of the BCAA catabolic pathway intermediates and enzymes. Detailed pathway of the enzymes and intermediates involved in the catabolism of the three BCAAs; valine, isoleucine and leucine. The enzymes and catabolic intermediates used for the purposes of this study has been demarcated with an asterisk (*) sign.

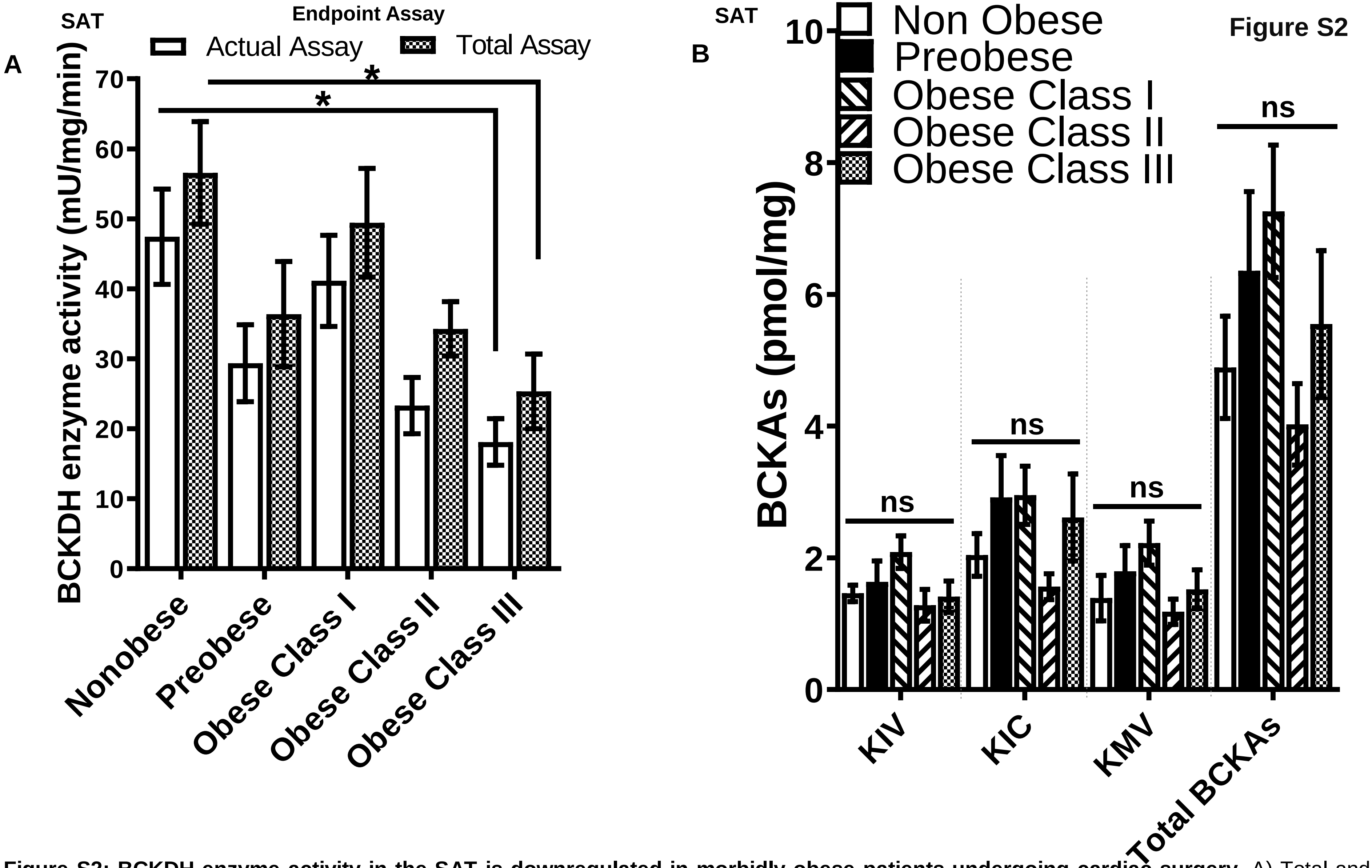

Figure S2: BCKDH enzyme activity in the SAT is downregulated in morbidly obese patients undergoing cardiac surgery. A) Total and actual BCKDH enzyme activity corrected to protein levels, measured at t=30min in the SAT of non-obese (n=5), pre-obese (n=6), class I obese (n=6), class II obese (n=6) and class III obese (n=6) in patients undergoing cardiac surgery. B) Intracellular BCKAs measured by UPLC MSMS in the SAT of non-obese (n=5), pre-obese (n=5), class I obese (n=5), class II obese (n=5) and class III obese (n=5) cardiac surgery patients. Statistical analysis was performed using a two-way ANOVA followed by a Tukey's multiple comparison test; *p <0,05, **p < 0.01, **** p <0.0001.

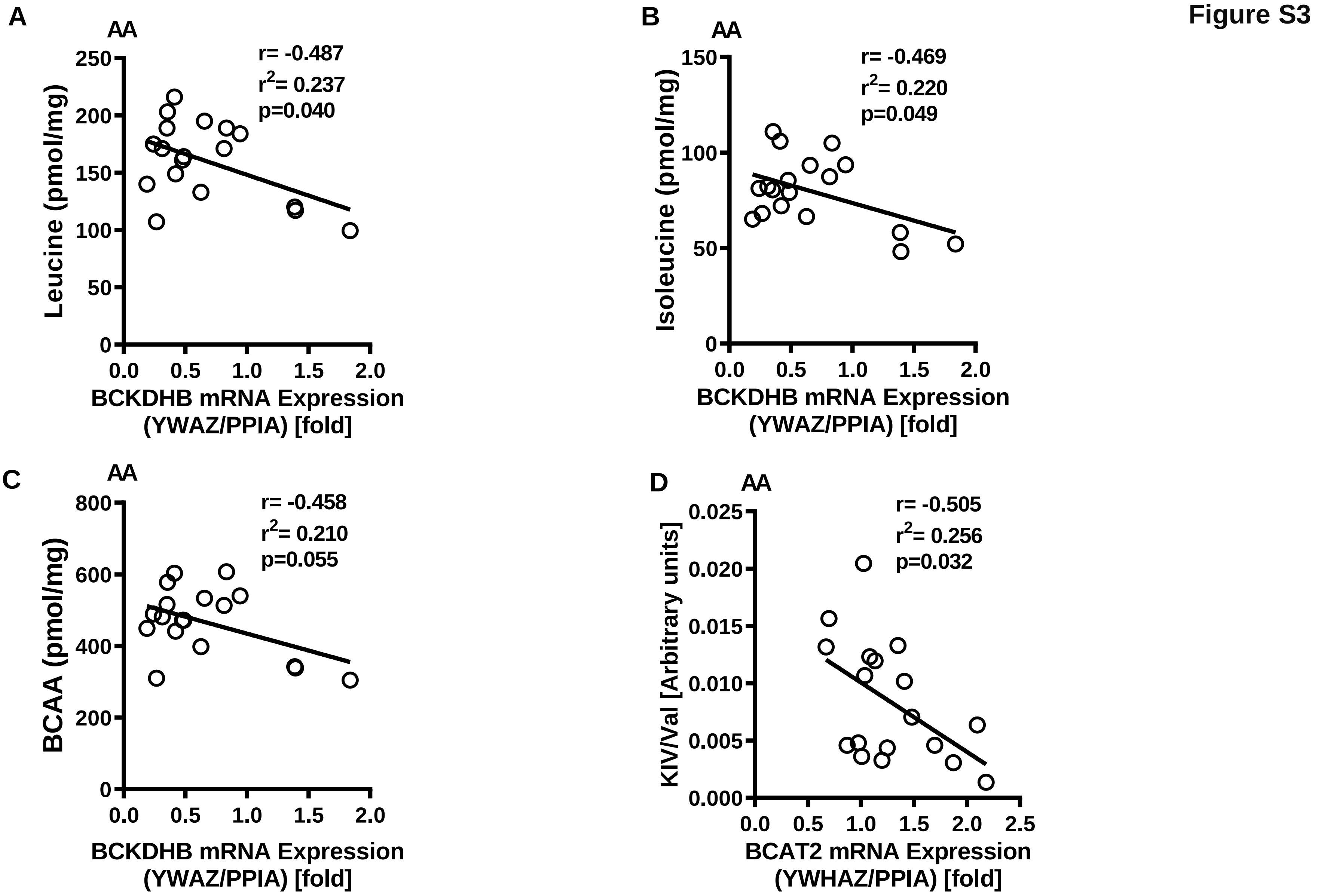

Figure S3: Intramyocellular BCAAs are correlated with mRNA levels of key BCAA metabolizing enzymes in AA tissues. Linear regression of A) leucine, B) isoleucine and C) total BCAAs correlated with BCKDHB mRNA levels. D) Linear regression of KIV to valine levels in the AA tissues of non-obese (n=5), pre-obese (n=5), class I obese (n=5), class II obese (n=5) and class III obese (n=5) cardiac surgery patients correlated with BCAT2 mRNA levels in the AA. Statistical analysis was performed using one-way ANOVA; followed by a Tukey's multiple comparison test; *p<0.05 was considered significant.

Figure S4

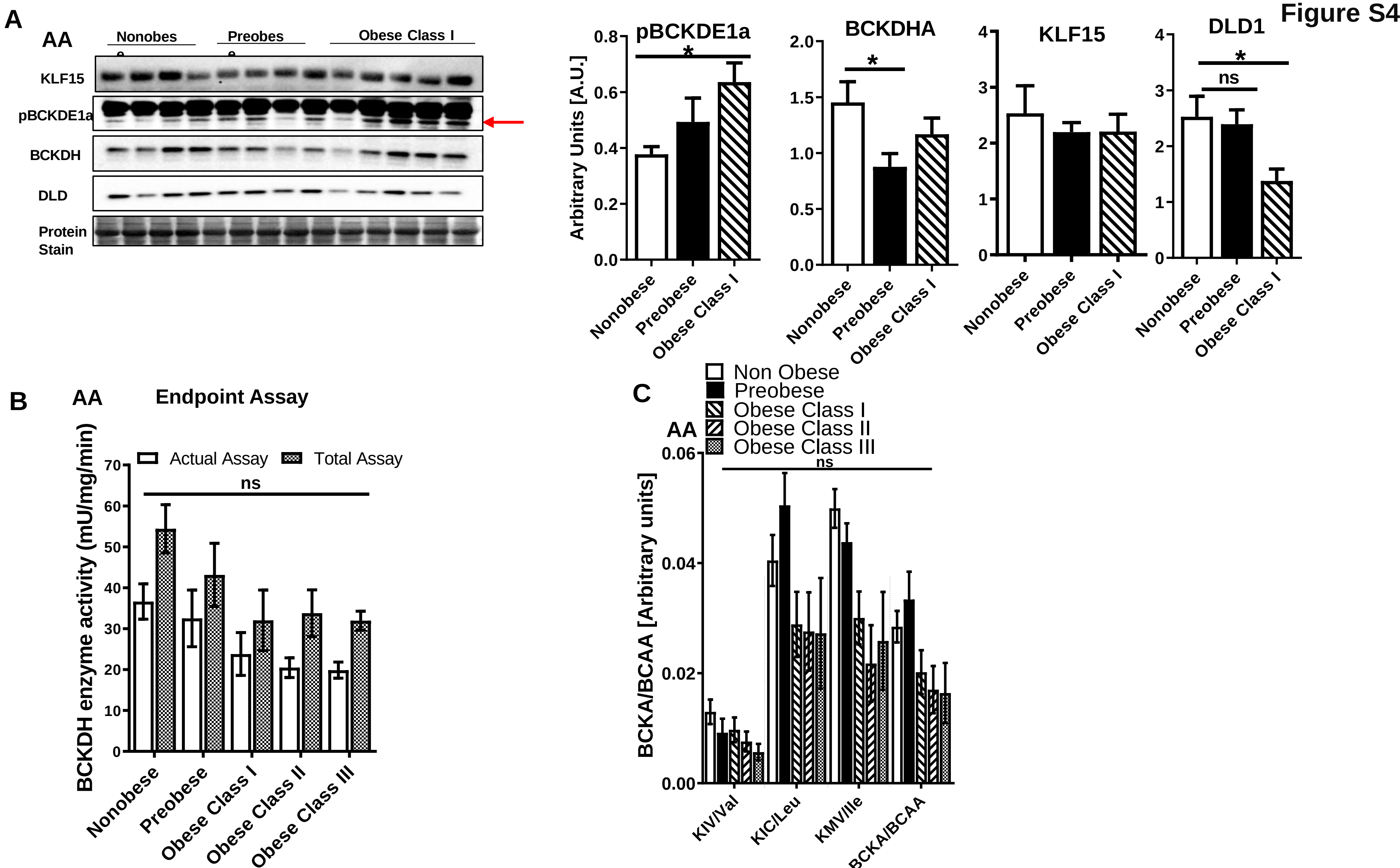

Figure S4: Changes in BCKDH enzyme activity and protein levels of BCAA catabolic enzymes independent of mRNA levels. A) Immunoblot and densitometric analysis of pBCKDE1a, BCKDH, DLD and KLF15 protein levels in the AA tissues of non-obese (n=4), pre-obese (n=4) and class 1 obese (n=5) cardiac surgery patients. Full length blots are presented in Figure S6. B) Total and actual BCKDH enzyme activity corrected to protein levels, measured at t=30min in the AA of non-obese (n=8), pre-obese (n=7), class I obese (n=6), class II obese (n=9) and class III obese (n=8) patients undergoing cardiac surgery. C) Ratio of BCKAs to BCAAs measured by UPLC MSMS in the AA of non-obese (n=5), pre-obese (n=5), class I obese (n=5), class II obese (n=5) and class III obese (n=5) cardiac surgery patients. Statistical analysis using a two-way ANOVA followed by a Tukey's multiple comparison test; *p <0,05, **p < 0.01, **** p <0.0001 as indicated.
